# Repulsive cues combined with physical barriers and cell–cell adhesion determine progenitor cell positioning during organogenesis

**DOI:** 10.1038/ncomms11288

**Published:** 2016-04-18

**Authors:** Azadeh Paksa, Jan Bandemer, Burkhard Hoeckendorf, Nitzan Razin, Katsiaryna Tarbashevich, Sofia Minina, Dana Meyen, Antonio Biundo, Sebastian A. Leidel, Nadine Peyrieras, Nir S. Gov, Philipp J. Keller, Erez Raz

**Affiliations:** 1 Institute for Cell Biology, ZMBE, Von-Esmarch-Street 56, 48149 Muenster, Germany; 2 Howard Hughes Medical Institute, Janelia Research Campus, 19700 Helix Drive, Ashburn, Virginia 20147, USA; 3 Department of Chemical Physics, Weizmann Institute of Science, Rehovot 76100, Israel; 4 Germ Cell Development, Max-Planck Institute of Biophysical Chemistry, Am Fassberg 11, 37070 Göttingen, Germany; 5 Max Planck Research Group for RNA Biology, Max Planck Institute for Molecular Biomedicine, Von-Esmarch-Strasse 54, 48149 Muenster, Germany; 6 USR3695 BioEmergences, CNRS, Université Paris-Saclay, Avenue de la Terrasse, 91190 Gif-sur-Yvette, France

## Abstract

The precise positioning of organ progenitor cells constitutes an essential, yet poorly understood step during organogenesis. Using primordial germ cells that participate in gonad formation, we present the developmental mechanisms maintaining a motile progenitor cell population at the site where the organ develops. Employing high-resolution live-cell microscopy, we find that repulsive cues coupled with physical barriers confine the cells to the correct bilateral positions. This analysis revealed that cell polarity changes on interaction with the physical barrier and that the establishment of compact clusters involves increased cell–cell interaction time. Using particle-based simulations, we demonstrate the role of reflecting barriers, from which cells turn away on contact, and the importance of proper cell–cell adhesion level for maintaining the tight cell clusters and their correct positioning at the target region. The combination of these developmental and cellular mechanisms prevents organ fusion, controls organ positioning and is thus critical for its proper function.

## Abstract (web summary)

The precise positioning of organ progenitor cells is essential for organ development and function. Here the authors use live imaging and mathematical modelling to show that the confinement of a motile progenitor cell population results from coupled physical barriers and cell-cell interactions.

## Graphical abstract

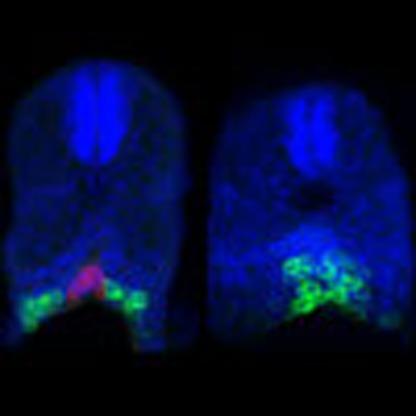

Organogenesis is a critical embryonic process, during which cells and tissues are organized to establish functional structures that carry out physiological roles during the life of the multicellular organism^1^. Indeed, abnormalities in this process can lead to severe pathological consequences (for example, organ fusion and malignancies associated with mismigrating cells^2^,^3^,^4^). A major challenge in developmental biology is thus to define the mechanisms that control cell positioning during organ formation to ensure its proper function (for example, ref. 5). The initial positioning of cells that form an organ is often controlled by guidance cues^6^,^7^ and by biophysical properties of the cells such as cell adhesion and surface tension^8^, which can involve the function of signalling molecules that regulate cell differentiation and behaviour^9^,^10^. Whereas the mechanisms that control cell migration have been extensively studied in the context of normal development and disease (for example, refs 11, 12, 13, 14), the mechanisms responsible for positioning and maintaining the cells at locations where organogenesis takes place are poorly understood. As an *in vivo* model for this process, we study gonad formation, focusing on stages immediately following the arrival of progenitor cells at the region where they participate in constructing the organ.

The gonad is composed of two cell populations, namely, germ cells and somatic cells that support the development of the germ cells into gametes^15^,^16^. In most organisms, germ cells are specified at early stages of development, and subsequently migrate to form two cell clusters on each side of the midline^11^. During this developmental stage, germ cells are referred to as primordial germ cells (PGCs).

The migration of the PGCs towards the region where the gonad develops typically occurs in close association with cells of endodermal origin and is directed by cues provided by somatic cells along the migration route^11^. Zebrafish (*Danio rerio*), mouse and chick PGCs are guided towards their target by the chemokine Cxcl12 that attracts cells expressing its cognate receptor, Cxcr4 (refs 17, 18, 19). In *Drosophila melanogaster*, a combination of repulsive and attractive cues guides the PGCs towards their targets (reviewed in ref. 11). Among those and relevant to this work, are repulsive cues generated by two lipid phosphate phosphatase (LPP) enzymes, termed Wunens (Wun and Wun2) (also termed as phosphatidic acid phosphatase type 2 (PPAP2)). The Wunens degrade phospholipid substrates^20^ and thereby direct *Drosophila* PGCs away from phospholipid-depleted domains^21^,^22^,^23^. Interestingly, Wunen substrates have been shown to regulate cell migration in other organisms as well^21^,^24^.

Following their arrival at the region where the gonad develops, the clustered PGCs remain at the position where they eventually interact with the somatic gonad precursor cells^11^. Despite the importance of this step, the mechanisms responsible for maintaining the PGC population in place, thereby allowing the later interaction with the somatic cells and the formation of a functional gonad, are currently unknown.

Here, we show that following arrival of PGCs at their migration target, the cells, although motile, form compact bilateral clusters as a result of different activities. First, we find that spatially restricted expression of zebrafish Wunen orthologs, LPP proteins, inhibits the movement of the cells towards the developing somites. Second, by employing live-cell imaging and mutant analysis, we show that the maintenance of separated arrangement of the PGC clusters critically depends on the interaction of this cell population with cells of the developing gut tissue that reside between them. Indeed, using a particle-based simulation to describe cell dynamics, we demonstrate that cell cluster size distribution and position, similar to that observed *in vivo,* can be attained by specific levels of cell–cell adhesion and tissue barriers from which cells are reflected. Together, we find that the first step in organ formation relies on the generation of domains in the embryo that are repulsive for cell migration, the presence of physical barriers, combined with preferential interaction among the cells. Collectively, these events restrict the progenitor cells to the region where the organ develops.

## Results

### Progenitor cells are motile following arrival at the target

**Figure 1:**
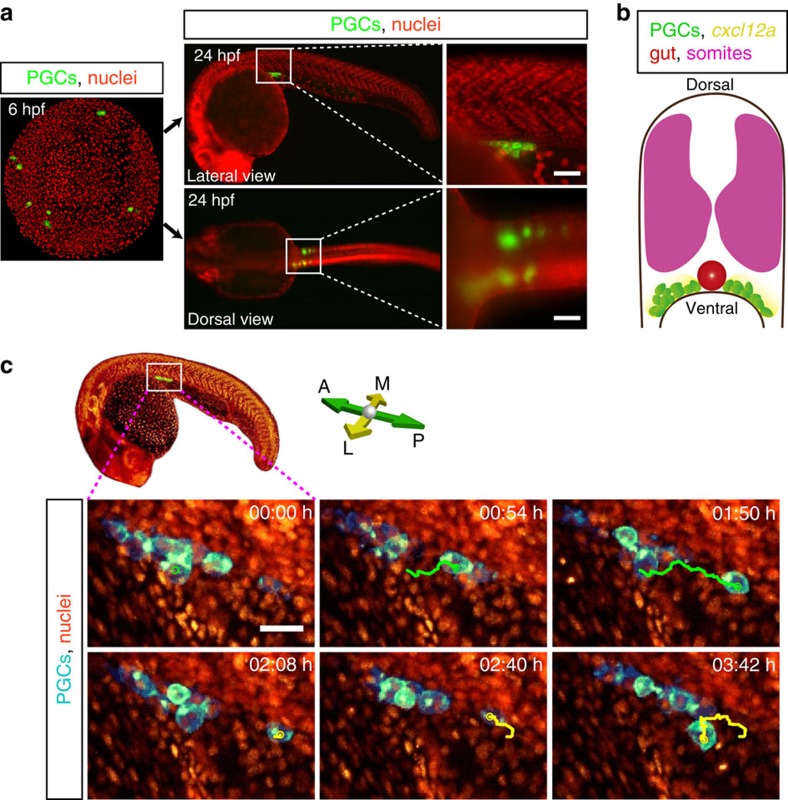
PGCs are motile at the gonad region. (**a**) PGCs migrate from four different positions in the embryo (green clusters in 6 hpf image) towards the developing gonads to form two separate cell clusters by end of the first day of embryonic development (lateral and dorsal views). Insets display higher magnification of the gonad region (white boxes). Scale bars represent 50 μm. (**b**) A schematic cross-section of a 1-day-old zebrafish embryo showing the somites (magenta), the two separate PGC clusters (green cells) located on either side of the developing gut (red structure), as well as the expression of *cxcl12a* at this stage (yellow). (**c**) Snapshots from a time-lapse movie (Supplementary Movie 2) showing a lateral view of a PGC cluster starting at 24 hpf In the first three time points posterior migration of a PGC is highlighted (green track) and lateral–medial migration of the same PGC is presented in the following panels (yellow track). Scale bar, 25 μm.

Following their specification at four locations (Fig. 1a, left panel), zebrafish PGCs migrate toward the regions where the gonads develop, forming two clusters separated by the developing gut and ventral to the somites by the end of the first day of embryonic development (Fig. 1a, right panels and Fig. 1b; reviewed in ref. 25). Importantly, similar to other organogenesis processes, the progenitor cells that reached their migration target maintain their position and participate in the establishment of functional organs, gonad in this case^1^.

A possible mechanism for retaining the PGCs at the site where the organ develops is loss of cell motility following arrival at the target. To examine PGCs behaviour following arrival at the gonad site, we employed advanced imaging tools to visualize the cells at this stage.

While monitoring the cells within clusters that are located at deep positions in the developing embryo, we detected strong active motility of PGCs relative to each other and with respect to neighbouring somatic cells (Supplementary Movies 1 and 2 for simultaneous multiview light-sheet (SiMView) microscopy^26^,^27^ and two-photon microscopy, respectively^28^, within 24–34 hours post fertilization (hpf) embryos; Fig. 1c; Supplementary Table 1 for experimental designs). Interestingly, while clearly motile, the speed of PGCs is strongly reduced on arrival at the gonad region (0.6 μm min^−1^; *n*=6 cells that did not contact other PGCs, imaged using SiMView Microscopy in three independent experiments), compared with their migration speed during early phases (2 μm min^−1^)^29^. Maintaining highly motile PGCs at the gonad site thus requires mechanisms to inhibit the migration of PGCs away from the region where the organ develops. This prompted us to explore the mechanisms acting to prevent PGCs from migrating dorsally into the somites and across the midline of the embryo to maintain the two separated cell clusters in their characteristic bilateral configuration.

### Guidance cue-independent positioning of progenitor cells

**Figure 2:**
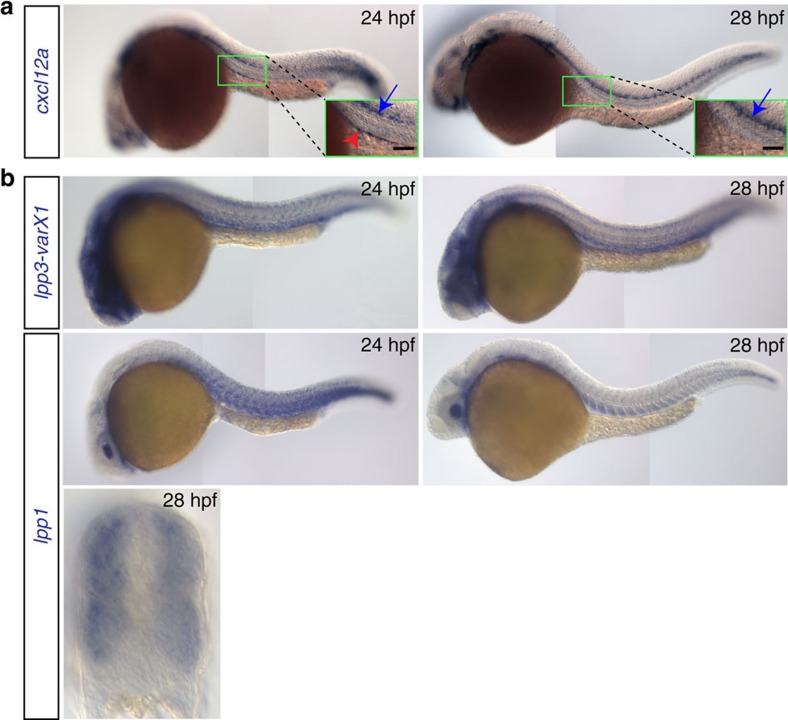
Expression patterns of *cxcl12a* and *lpp* variants. (**a**) *cxcl12a* is expressed at the site where the gonad develops within a 24 hpf embryo (green box and red arrowhead in the inset in the left panel), but not in 28 hpf embryos (right panel). Higher expression level of *cxcl12a* is detected in the lateral line (blue arrows). Scale bar, 50 μm. (**b**) *lpp1* (variants X1 and X2) and *lpp3* are expressed in the somites and developing vessels. See also Supplementary Fig. 3.

Zebrafish PGCs are directed towards their target by the attractant Cxcl12a that is produced by somatic cells along the migration route^17^,^30^. Considering that the PGCs are motile after arrival at the gonad region, a mechanism for confining the cells to this domain could involve continuous restricted expression of *cxcl12a* at the target area. However, whereas a low level of *cxcl12a* transcripts could still be detected at the gonad region in 24 hpf embryos (Fig. 2a, red arrowhead; Supplementary Fig. 1a, magenta bracket upper panel), the level of mRNA expression progressively decreased. Even when employing RNAscope^31^, a sensitive and quantitative method to detect RNA expression, no specific signal was detectable by 28 hpf, (Fig. 2a, right panel; Supplementary Fig. 1a, magenta bracket lower panel), while nearby structures showed very strong expression (for example, the lateral line that is located about 60 μm from the PGC cluster; Fig. 2a, blue arrow in the right panel; Supplementary Fig. 1a, white arrows). The lack of *cxcl12a* expression at the gonad region at 28 hpf, coupled with the absence of detectable transcripts of *cxcl12b* encoding for a weaker Cxcr4b ligand^32^ (Supplementary Fig. 1b; left panel) and the fact that the PGCs apparently do not respond to nearby strong sources of Cxcl12a, call for other mechanisms that would confine the cells to the site where the gonad develops.

### Control of progenitor cell migration by repulsive tissues

Keeping the PGC clusters at ventral locations where the gonads develop could be achieved by rendering the neighbouring tissues, such as developing somites, repulsive for the motile cells. An analogous scenario was described in the context of PGC migration in *Drosophila*. There, Wunen proteins were shown to convert tissues, in which they were expressed, into domains that were avoided by migrating PGCs^23^,^33^. We sought to determine whether Wunen orthologs in zebrafish control the localization of cell clusters. To this end, we identified and analysed 6 LPP orthologs (LPP1, LPP1-like, LPP2, LPP2-like, LPP3 and phosphatidic acid phosphatase type 2d) that exhibit conservation of the phosphatase domain to that of *Drosophila* and human LPPs (Supplementary Fig. 2; refs 20, 33, 34). Interestingly, the mRNAs encoding for the zebrafish LPP proteins are expressed at the relevant stages in the somites and the developing vascular system, tissues located dorsally to the forming PGC clusters (Fig. 1a,b, Fig. 2b and Supplementary Fig. 3). Importantly, based on RNA-sequencing (RNA-seq) analysis, PGCs express the receptors for the LPP substrates (S1P and LPA) before (7 hpf) and after (36 hpf) arrival at the gonad region (Supplementary Table 2). The sequence similarity between zebrafish LPPs and the *Drosophila* Wunen proteins, along with the expression of the corresponding zebrafish mRNAs dorsal to the PGC clusters, prompted us to further probe their potential role in controlling cluster positioning. To investigate the function of these enzymes in the context of PGC migration, we conducted a set of loss- and gain-of-function experiments.

**Figure 3:**
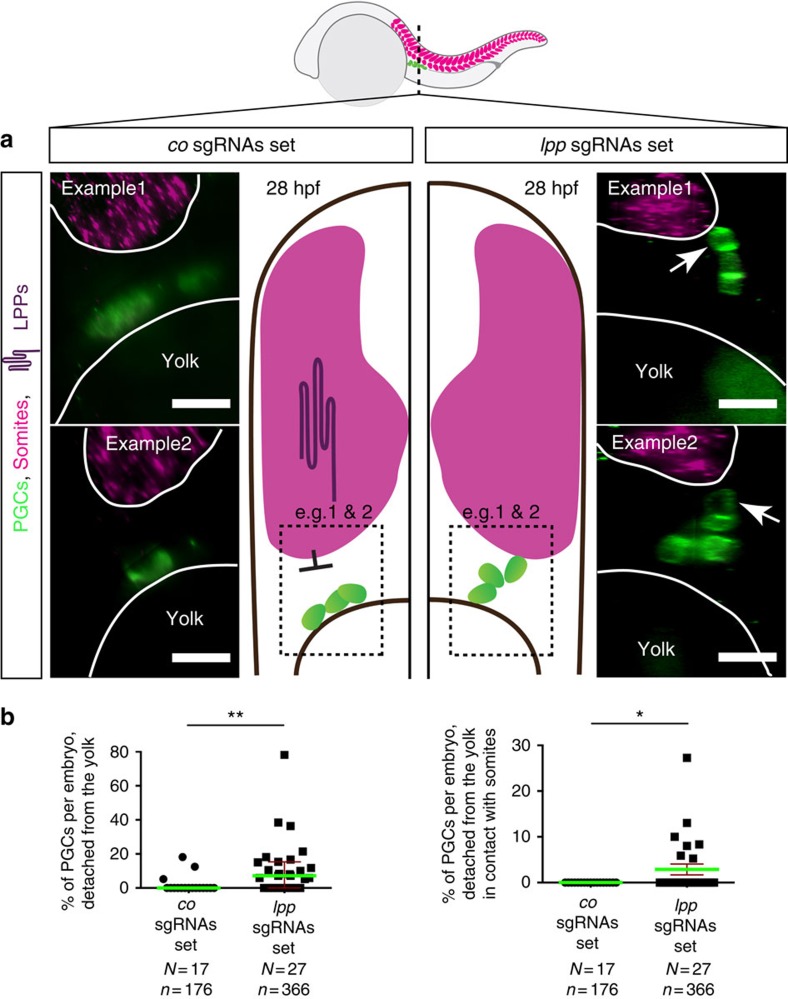
Abnormal positioning of PGCs in embryos treated with Cas9 and sgRNAs set against *lpp*s. (**a**) Optical cross-sections (plane marked by dashed line in the embryo scheme) of whole-mount 28 hpf embryos (*Tg(kop:egfp-f-3′nos3*) expressing EGFP in their PGCs following RNAscope procedure labelling *myoD* expression in somites (magenta, border marked in white). In contrast with control embryos (left panels), in embryos treated with Cas9 and a set of sgRNAs targeting 6 *lpp*s (right panels) PGCs detach from the yolk and can contact somites (arrows). Scale bars, 20 μm. Dorsal is up. (**b**) The percentages of PGCs per embryo detached from the yolk (left graph) and percentage of those in contact with the somites (right graph) is significantly elevated in LPPs-depleted embryos. The statistical significance was evaluated using the Mann–Whitney *U*-test (*P≤0.05, **P≤0.01). Green lines signify the mean, error bars the standard error of the mean (s.e.m), N the number of embryos and n the number of PGCs examined.

We employed a G0 CRISPR/Cas9-based genome-editing procedure^35^,^36^,^37^ to simultaneously knockout the six LPP-encoding genes and to explore the relevance of LPP function for PGC positioning at the gonad region (see Supplementary Table 3 for the sequences of the single guide RNAs (sgRNAs) and Supplementary Fig. 4 for guide activity assays). In embryos treated with a set of control sgRNAs (targeting the *tyrosinase*, *albino* and *golden* genes required for pigmentation, each by four sgRNAs), the PGCs (green-labelled cells in Fig. 3a) were in contact with the yolk and distant from the border of the somites (magenta label, outlined in white in Fig. 3a). Strikingly, in embryos treated simultaneously with multiple sgRNAs against the six *lpp* genes (each gene targeted by several sgRNAs), a significant number of PGCs were located at positions distant from the yolk (Fig. 3a and Fig. 3b left), while some of those contacted the somites (arrows in Fig. 3a and Fig. 3b right). Importantly, these phenotypes were observed in embryos that otherwise developed normally (Supplementary Fig. 5a).

To independently verify these results, we simultaneously inhibited the expression of all LPP proteins using morpholino antisense oligonucleotides (MO) (Supplementary Fig. 6; see Supplementary Table 4 for MO used). Similar to the results obtained in the CRISPR/Cas9-based experiment, PGCs detached from the yolk and were positioned closer to the somites (Supplementary Fig. 7, assayed in “class1 embryos” presented in Supplementary Fig. 5b). Interestingly, consistent with these results, migration of PGCs away from the yolk was observed in the case of *spadetail* mutants, in which somitic mesoderm does not differentiate properly, although the basis for this phenotype was not known then^38^.

Collectively, the phenotypes observed when reducing the function of LPPs are consistent with the idea that the function of these enzymes in the somites restricts the PGCs to ventral locations. As suggested by the distance between PGCs and the LPP-expressing somites, the effective range of the repulsive activity in wild-type embryos is 15–30 μm (Fig. 3a *co* sgRNAs; Supplementary Fig. 7a, *co* MO antisense oligonucleotides), similar to that determined in *Drosophila* (33 μm; ref. 39).

**Figure 4:**
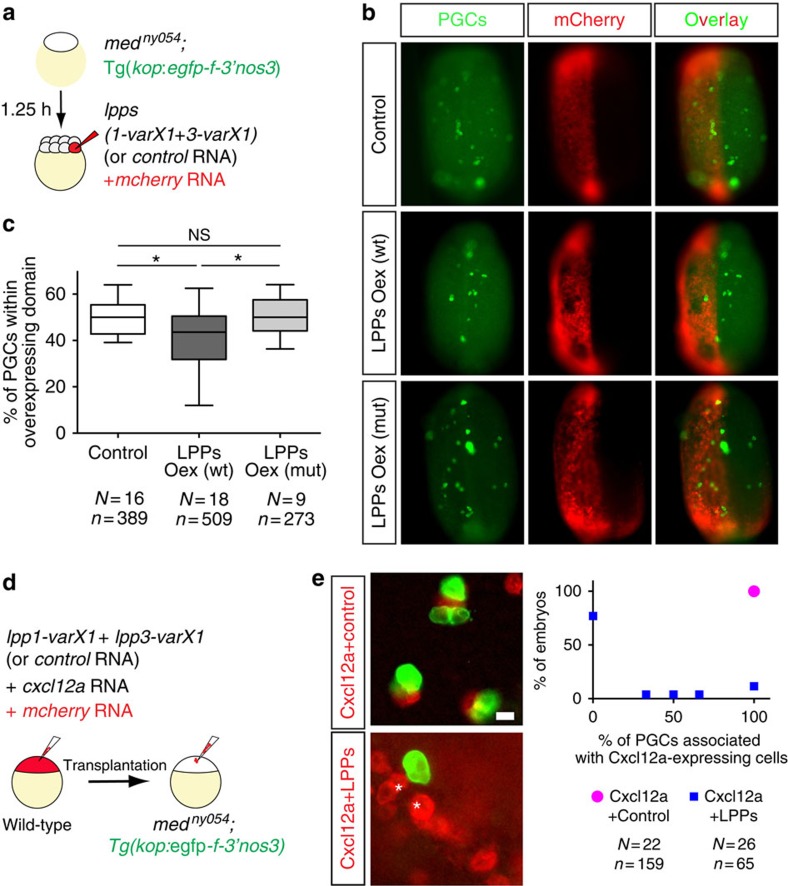
PGCs avoid regions expressing LPP proteins. (**a**) Generation of embryos lacking Cxcl12a, whose PGCs are labelled by EGFP and overexpress either LPP proteins or a Control protein in mCherry-labelled half of the embryo. (**b**) PGCs avoid cellular domains of the embryos, which overexpress LPPs (middle row), as compared to control domains (top row) or those overexpressing phosphatase-inactive versions of LPPs (lower row). (**c**) A significant reduction in the percentage of PGCs located within the LPPs-overexpressing domain in 10 hpf embryos (one-way analysis of variance; *P≤0.05). Error bars designate minimum to maximum range of the data points. *N* and *n* show the number of embryos and PGCs, respectively. See also Supplementary Fig. 8. (**d**) mCherry-labelled cells overexpressing Cxcl12a with either a Control Protein or LPPs were transplanted into embryos lacking Cxcl12a (*med*^*ny054*^) whose PGCs express EGFP. (**e**) Images and a graph demonstrating the association of PGCs with Cxcl12a-expressing cells in control embryos (upper image, magenta point in graph) and the lack of interaction with cells expressing Cxcl12a and LPPs (starred red cells in lower image, blue points in graph, 77% of embryos showed absolutely no cell association). The statistical significance was evaluated using the Mann–Whitney *U*-test (****P≤0.0001). Scale bar, 15 μm. See also Supplementary Movie 3 and Supplementary Fig. 9.

To complement the loss-of-function experiments, we sought to determine whether LPPs expression is sufficient for transforming embryonic structures into less favourable regions. To this end, we generated embryos lacking the guidance cue Cxcl12a, in which certain domains were engineered to overexpress the LPP proteins or a control protein (Fig. 4a and red-labelled regions in Fig. 4b). LPP1-varX1 and LPP3-varX1 that are highly expressed in the tissues dorsal to PGC clusters and exhibit the highest similarity to the *Drosophila* Wunen proteins were tested in these experiments. Remarkably, we observed a significant bias in PGC localization, as PGCs (green-labelled cells in Fig. 4b) were less likely to reside within domains overexpressing LPPs (Fig. 4b, middle row and Fig. 4c). Similar results were obtained in embryos lacking both guidance cues Cxcl12a and Cxcl12b (Supplementary Fig. 8a). This bias was not detected when the overexpressed protein was irrelevant for the process, nor when the overexpressed LPPs were mutated in their active sites (Fig. 4b, upper and lower rows respectively; Fig. 4c; black boxes in Supplementary Fig. 2 that mark mutation sites^24^). It is noteworthy that the total number of PGCs in the different experiments remained unchanged (Supplementary Fig. 8b), providing evidence that the observed bias in cell localization resulted from an effect on cell behaviour, rather than enhanced PGC death within domains of LPPs overexpression.

To characterize the cellular behaviour of PGCs at high resolution on encountering LPP-expressing cells, we engineered somatic cells to express the chemoattractant Cxcl12a together with LPPs or a control protein (Fig. 4d). As expected, PGCs directionally migrated towards Cxcl12a-expressing cells (Supplementary Fig. 9) and in all cases remained in close association with them (Fig. 4e, upper panel and graph; Supplementary Movie 3). In a marked contrast, however, PGCs were rarely attracted towards cells co-expressing LPPs and Cxcl12a (Supplementary Fig. 9). Importantly, in most embryos (77%) PGCs that did approach LPPs- and Cxcl12a-expressing cells were unable to establish a stable association with them (Fig. 4e, lower panel and graph; Supplementary Movie 3).

Altogether, PGCs are rarely attracted to LPPs- and Cxcl12a-expressing cells, and if located in their vicinity, they do not remain associated with them. Accordingly, these findings suggest that the activity of LPP enzymes in the tissues located dorsal to the PGC clusters is responsible for generating cellular domains that PGCs actively avoid, thereby confining the cell clusters to ventral locations and preventing them from reaching domains where Cxcl12a is expressed (for example, the lateral line, Fig. 1b and Supplementary Fig. 1a).

### Separation of the motile cell clusters by the developing gut

**Figure 5:**
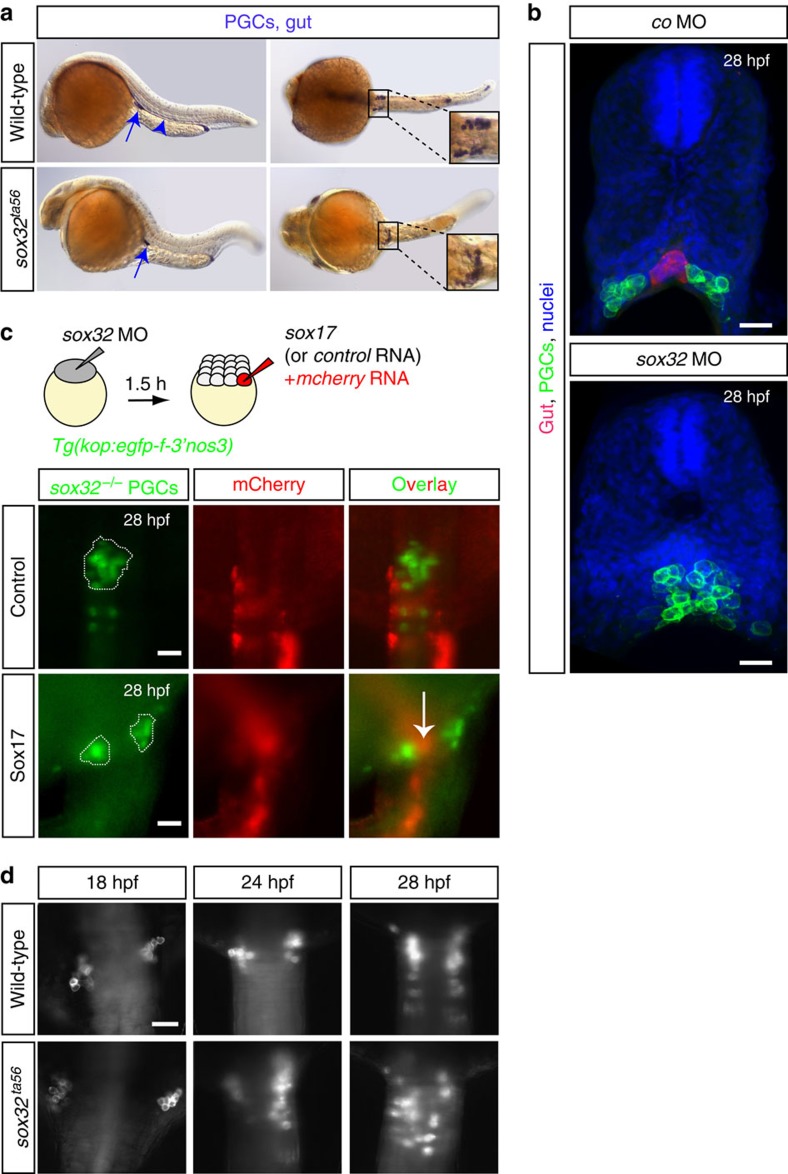
Lack of the developing gut causes PGC cluster fusion. (**a**) Whole-mount *in situ* hybridization on 30 hpf wild-type (*N*=420) and *sox32* mutant embryos (*N*=89). PGCs are labelled with *nanos3* (*nos3*, arrows) and the gut with *foxa3* probe (arrowhead, missing in *sox32* mutant embryos), both in blue. Unlike the separated PGC clusters in wild-type embryos, clusters are fused at the midline in *sox32* mutant embryos (upper and lower panel images, respectively). Lateral (left panels) and dorsal (right panels) views are shown. (**b**) Cross-sections of 28 hpf *sox17:dsred* transgenic embryos whose gut is labelled in red and the PGCs membrane with EGFP. In control embryos (upper panel; *N*=5) bilateral PGC clusters form on either side of the gut tube. In embryos lacking the gut (Sox32-deficient; *N*=4) the PGC clusters fuse (lower panel). Nuclei counterstained with Hoechst. Scale bars, 25 μm. (**c**) Generation of mosaic embryos lacking Sox32 function in all cells whose endoderm is restored by providing Sox17 function to a group of cells (Scheme). The Sox32-deficient PGC clusters (green) are separated by the gut tissue at 28 hpf (red in lower panel, arrow; *N*=13), while in control embryos lacking endodermal tissues fused PGC clusters are observed (upper panel; *N*=24). Anterior is up. (**d**) PGC clusters in wild-type (*N*=20) and *sox32* mutant (*N*=7) embryos lacking the gut tissue at 18, 24 and 28 hpf showing the dynamics of the fusion. *N* is the number of embryos analysed. Scale bar, 50 μm. Anterior is up.

Maintaining a bilateral organization of separated PGC clusters following arrival of the cells at the region where the gonad develops could in principle be achieved by the presence of medial structures that would prevent PGC migration towards the midline. We addressed this possibility by examining the positioning of the cell clusters in embryos lacking different midline structures. We found that in embryos lacking the vasculature or the notochord, the positioning of the PGC clusters was not affected (in *cloche* and *floating head* mutant embryos respectively, Supplementary Fig. 10). Conversely, a complete fusion between the two cell groups was invariably observed in embryos in which endoderm development was blocked, such that the developing gut was not positioned in between the two PGC clusters (that is, in *casanova* mutants lacking the function of the Sox32 protein^40^,^41^, Fig. 5a,b).

While the results presented above highlight what appears to be a novel role for endodermal tissues in maintaining the position of the developing gonad, the endoderm in other organisms is involved in earlier stages of PGC migration (for example, in *Drosophila* and the mouse^11^,^42^). It is thus formally possible that the PGC cluster fusion in zebrafish embryos lacking the gut could arise from early abnormal PGC development or migration. However, PGCs remained in close proximity to the yolk in embryos that lack endoderm, similar to their position in wild-type embryos, where PGCs are intercalated among endodermal cells and both cell types reside directly on top of the yolk syncytial layer (YSL) (Supplementary Fig. 11). Consistently, PGCs in Sox32-deficient embryos appeared to be properly specified and to develop normally as judged by marker gene expression (*nos3* and *piwil1*) and the presence of PGC-specific structures (for example, germ cell granules; Supplementary Fig. 12). Thus, endoderm development does not appear to bear on the specification, the early development and the migration of the PGCs, until they reach the region where the gonad develops. We infer from these results that endodermal cells represent a late requirement for maintenance of the separated arrangement of cell clusters, rather than a requirement for *Sox32* gene within the PGCs (Supplementary Table 5 for *sox32* expression). Indeed, restoring gut formation in Sox32 knocked-down embryos by injection of the *sox17* RNA (encoding for the Sox17 protein that acts downstream of Sox32 (ref. 43)) in one of the cells at the 16-cell stage that only contributes to somatic lineages, reversed the PGC cluster fusion phenotype (Fig. 5c).

Importantly, we did not observe any global defect in the patterning of the neighbouring mesodermal tissues (for example, pronephric ducts and the glomeruli, green arrows in Supplementary Fig. 13a–b) in embryos deficient for endodermal development, defects that could serve as the basis for the strong PGC cluster fusion phenotype. Interestingly, however, in Sox32 knocked-down embryos, the separated *cxcl12a* expression domains visible at 15 and 18 hpf are fused in 24 hpf embryos, while *cxcl12a* expression progressively declines and is not detectable at 28 hpf (Supplementary Fig. 13c). In those developmental stages the expression of the other *cxcl12* molecule, *cxcl12b*, could not be detected in this area at 24 hpf neither in control nor in Sox32 knocked-down embryos (Supplementary Fig. 1b). These results define a novel role for the developing gut in controlling the distribution of Cxcl12a around the time PGCs arrive at their target. We conclude that the fusion of the cluster in *sox32* mutant embryos is likely to result from the abnormal Cxcl12a expression pattern and the presence of a region free of endodermal cells at the midline. In wild-type embryos, however, maintaining the separation of the two clusters of motile cells during the following stages when *cxcl12a* expression is diminished (Fig. 2a and Supplementary Fig. 1a, 28 hpf images) could solely depend on the gut that develops at this position.

### The developing gut acts as a physical barrier for PGCs

To characterize the role of the developing gut in maintaining the separation between the PGC clusters, we assessed the position of the cells at different time points following their arrival at the gonad region. Whereas PGC clusters were positioned in similar locations in wild-type and gut-deficient embryos at 18 hpf, in *sox32*^*ta56*^ embryos the clusters progressively came closer to one another (for example, at 24 hpf) and eventually fused at the midline of embryos lacking the gut tube (at 28 hpf; Fig. 5d).

**Figure 6:**
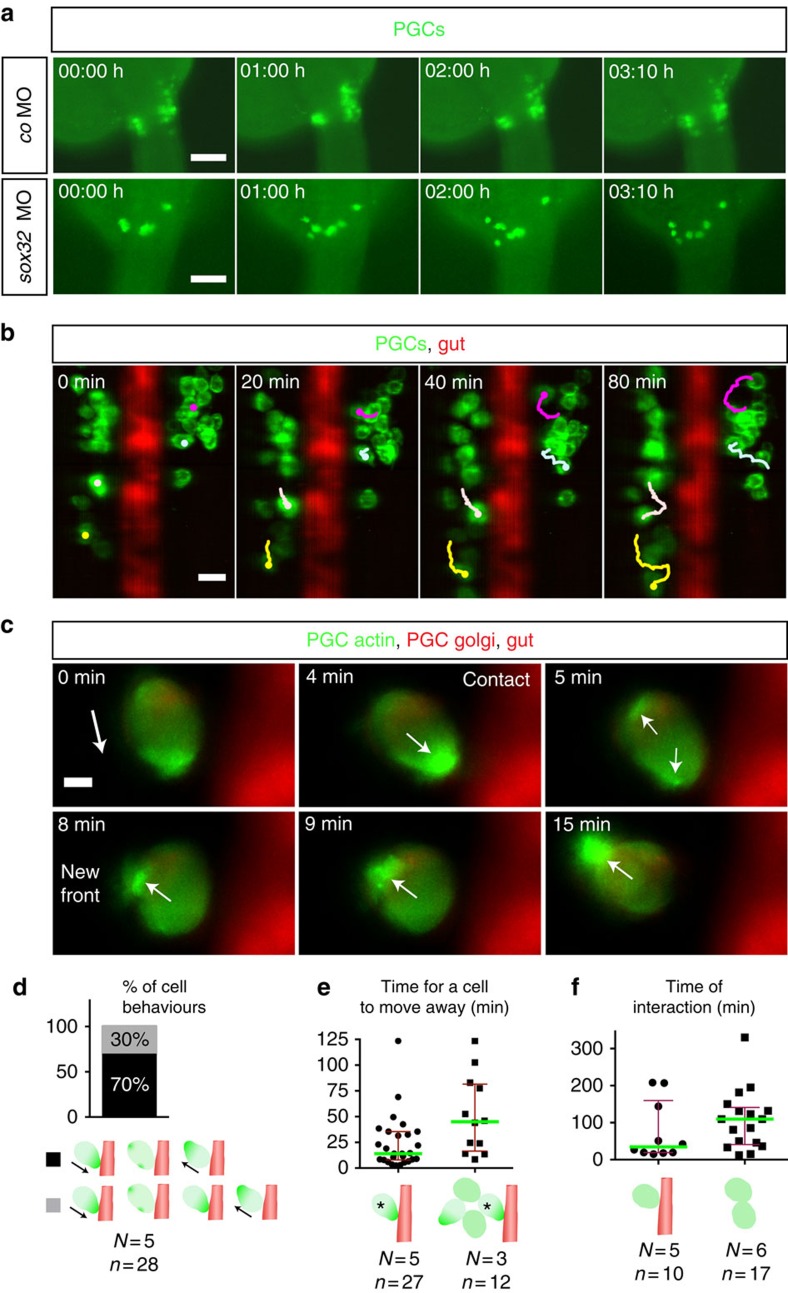
The developing gut functions as a physical barrier. (**a**) PGCs exhibiting dynamic movements within separated clusters (control, upper), while in embryos lacking the gut (lower panels) PGCs migrate over the midline to form one cluster (Supplementary Movie 4). Time point 0 corresponds to 24.5 hpf Scale bars, 100 μm. (**b**) Four representative migration tracks of PGCs relative to the gut (Supplementary Movie 5). PGC tracking using ImageJ. Scale bar, 25 μm. (**c**) Interaction of a PGC with the gut tube (Supplementary Movie 6). Polarity change in actin distribution is observed on contact. Time point 0 corresponds to 25 hpf The white arrow displays the direction of actin polarity. Scale bar, 5 μm. (**d**) PGC behaviours. On touching the gut, the main behaviour observed (29/42 encounters for 28 PGCs) is a rapid (14 min) polarity inversion away from the barrier and a change in the direction of migration. In the remaining encounters (13/42) PGCs exhibited a prolonged contact with the gut (69 min) without stable polariziation. (**e**) Cell crowding prolongs the time required for moving away from the barrier (45 versus 14 min; Mann–Whitney *U*-test, **P≤0.01). (**f**) Increased interaction time among PGCs that do not touch (right), as compared with the interaction time large and small clusters of the PGCs with the barrier (left). (**d**–**f**) Error bars display interquartile range, green lines median values, *N* and *n* number of embryos and PGCs respectively. The PGCs are shown in green and the gut in red (gut not presented in the right schematic drawing in **f** where PGC–PGC interaction time is displayed).

To determine the basis for the observed phenotype, we visualized the PGCs in live embryos at the relevant developmental stages (between 24 and 28 hpf). Remarkably, similar to PGCs in wild-type embryos, PGCs in *sox32*^*ta56*^ embryos exhibited prominent, active motility following arrival at the gonad region, which in this case was observed at the midline of the embryo (Fig. 6a; Supplementary Movie 4). These findings suggest that the arrival of the PGCs at ectopic positions in gut-deficient embryos results from active migration.

To examine the role the gut tube plays in patterning the gonad, we followed the position of the PGCs in wild-type embryos relative to the developing gut at the clustering region employing SiMView microscopy^27^. Notably, PGCs changed their direction of migration and moved away from the endodermal tube on contact (Fig. 6b; Supplementary Movie 5). To characterize the dynamic behaviour of the migrating PGCs on interaction with the gut, we followed the cells and the polar distribution of actin within them at high-resolution. Strikingly, the most common behaviour PGCs exhibited (70% of the cases, Fig. 6d) after contacting the gut was a rapid ‘flip' in polarity (Fig. 6c). Specifically, the enrichment of actin at the contact site at the cell front was followed by the simultaneous formation of minor actin-rich structures at the side and the back of the cell (Fig. 6c; Supplementary Movie 6). This rapid polarity change was followed by the establishment of a new cell front at the previous rear of the cell and migration of the PGC away from the developing gut (Fig. 6c,d; the 14 min median in Fig. 6e; Supplementary Movie 6). The remaining fraction of the behaviours (30%, Fig. 6d) was characterized by a longer time period (69 min) for establishment of the polarity at the new front after touching the gut tube (Fig. 6d). It is noteworthy that the rapid versus the prolonged time required for cell polarity change does not result from differences among the PGCs, since single PGCs can exhibit both behaviours. The change of cell polarity and migration direction of PGCs on touching the gut, coupled with the observation that 30% of the cell behaviours involved prolonged contact with the gut indicate that the developing gut constitutes a physical barrier, rather than a tissue that produces repulsive cues. Visualizing the actin-rich cell front, we could, for the first time describe the dynamics of polarity changes in a migrating cell on interaction with a physical barrier *in vivo*. Interestingly, when cells were surrounded by other PGCs, a longer time was required for the cell to find a path free of both PGCs and the gut to migrate away from it (the 45 min median in Fig. 6e; Supplementary Movie 7), indicating that intra-cluster interactions contribute to the observed migration patterns.

Altogether, our analyses of cell behaviour and polarity highlight the role of endodermal tissue in separating the progenitor germ cell clusters of the developing gonad.

### Reflective boundaries and cell adhesion maintain clusters

**Figure 7:**
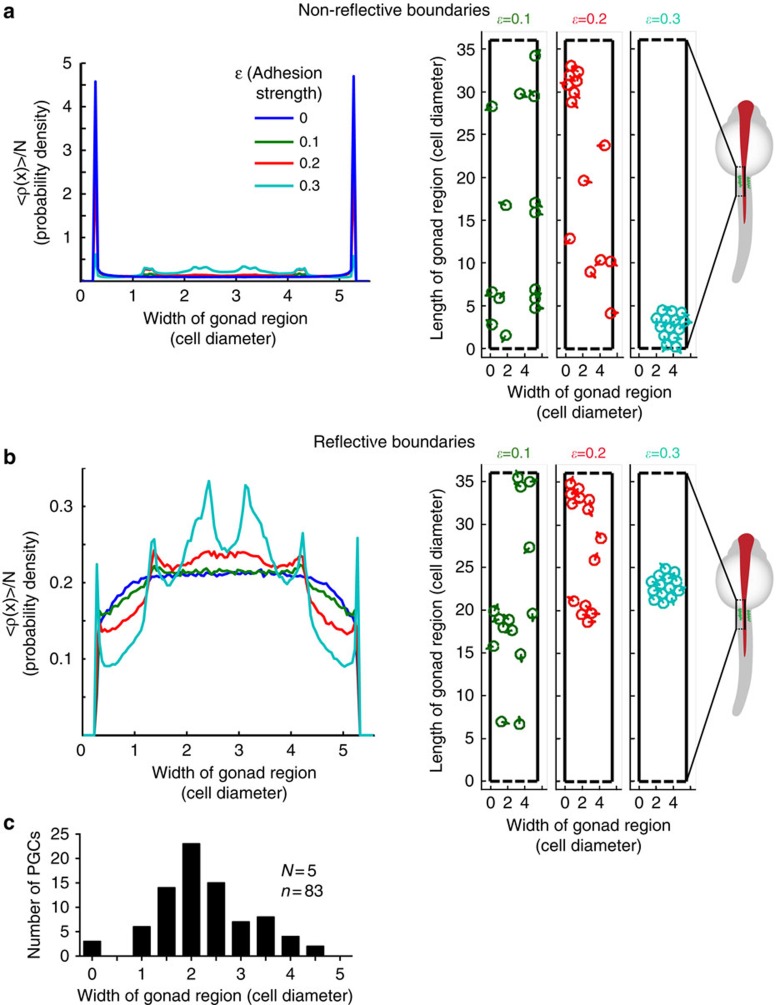
Boundary conditions and cell–cell adhesion level control cell cluster size and positioning. The steady-state distribution of 14 particles across the gonad region (black box in the schematic zebrafish embryo; five cell diameter wide) that is confined by non-reflective (**a**) or reflective boundaries (**b**) for different cell–cell adhesion levels (*ɛ*=0–0.3 (a.u.)). The y axes in the graphs represent the probability density to find a given particle at a certain position at the site of gonad. Snapshots from Supplementary Movies 9–10 (*t*=4850, min) for different *ɛ* values are provided on the right in **a** and **b**, respectively. (**c**) The distribution of cells at the gonad site in 24–25 hpf zebrafish embryos. *N* is the number of embryos and *n* that of PGCs.

To investigate whether differences between PGC–PGC and PGC–gut interactions could play a role in cluster formation and maintenance, we first compared the durations of such events. Interestingly, at the time of cell clustering, PGCs interact with each other for an extended time (109 min), as compared with the average duration of the PGC–gut contact (34 min; Fig. 6f; Supplementary Movie 8, sections 2 and 3). These observations suggest that PGCs maintain contact on interaction, a behaviour not observed on interaction with the gut. To quantitatively study the effects of these cellular features on the generation and positioning of cell clusters within the gonad region, we employed a simple (minimal) model, using two-dimensional particle-based simulations (Fig. 7a,b; Supplementary Movies 9–10; see methods section for details). In these simulations, the number and size of cells, their velocity, rate of change in migration direction and the dimensions of the region, as experimentally measured, were used. Each cell is represented by a particle that is interacting with the other particles (cells), with the up–down boundaries in periodic channel geometry and with the left–right boundaries as described below. The particles in the model are point-like objects that exert mutual attraction at a defined distance between them (representing cell–cell adhesion). A repulsive core prevents the particles from occupying the same position when compressed against each other. This attractive isotropic interaction potential between cells is quantified by the parameter *ɛ* in the model. The dynamics of the particles obeys persistent random walk behaviour^44^, whereby free self-propelled particles move at a constant velocity of 0.6 μm min^−1^, while the direction of motion is diffusing randomly with a rotational diffusion coefficient of *D*_*r*_=1/(60 min). The path of such a particle maintains directional persistence for an average duration of 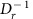. For simplicity, we modelled the region where the gonad develops as a chamber consisting of rigid left–right boundaries, which cells cannot penetrate. We considered two possible scenarios following cell contact with the boundaries: (i) the direction of cell motility is unaffected (non-reflective boundary), and (ii) the direction of motion of the cells is immediately changed to move perpendicularly away from the boundaries (reflective boundary). The reflective boundary acts on the cells in the same manner as contact inhibition of locomotion^45^,^46^. For both of these scenarios, we examined how altering the cell–cell adhesion strength (*ɛ*) affected the generation and positioning of cell clusters within the chamber.

We plotted the spatial density of the particles across the width of the channel for non-reflective boundaries (Fig. 7a) and for reflective boundaries (Fig. 7b). We found that in the absence of reflective boundaries and at lower adhesion levels (*ɛ*<0.3), a significant proportion of the cells are positioned adjacent to the walls (Fig. 7a; Supplementary Movie 9), which is a previously described effect observed in self-propelled particle systems^47^,^48^. Importantly, accumulation of PGCs along the borders of the gonad region is not observed in wild-type embryos (Fig. 7c). On the basis of our simulations, high levels of cell–cell adhesion result in the accumulation of cells in a single cluster positioned at the centre of the channel, regardless of the properties of the boundaries (*ɛ*=0.3 adhesion level in Fig. 7a,b and Supplementary Movies 9–10). Such single clusters that contain all the cells at the gonad region are, however, not observed in embryos. This suggests that while adhesion can promote cluster formation, its level should be moderate to facilitate cluster size distribution similar to those observed *in vivo* (large and small clusters, *ɛ*=0.2, Supplementary Fig. 14 and Supplementary Movie 1). Next, we quantitatively assessed the effect of adhesion levels (*ɛ*) and the boundary conditions on cluster size distribution and compared the model-predicted values with those measured experimentally. Interestingly, we find that the experimentally observed fraction of cells in large clusters is achieved using *ɛ*≈0.2, and is weakly dependent on the boundary condition (Supplementary Fig. 14). Importantly, however, under moderate adhesion (*ɛ*≈0.2), the accumulation of cells at the centre of the channel (manifested by a low variance of the positions of cells across the chamber), as observed *in vivo*, can only be achieved when reflective boundaries exist (Fig. 7a,b, Supplementary Fig. 15, Supplementary Movie 10). In contrast, central positioning of cell clusters for non-reflective boundaries is similar to the measured value only for adhesion levels larger than 0.3 (an adhesion level that results in cluster sizes that do not match the experimentally measured values, Supplementary Fig. 15). A detailed calculation of the cell position distribution in the case of no interactions among the cells is provided in the Supplementary Note.

Collectively, the theoretical model highlights the significance of physical barriers acting as reflective boundaries in cell cluster positioning. In addition, we infer from our simulations that PGCs should possess a particular level of homotypic cell–cell adhesion for proper cluster size distribution. Indeed, as compared with early migration stages, the expression level of several cell adhesion molecules is elevated in the PGCs at 36 hpf (Supplementary Table 6).

The proper progression of the events characterized above, that is, the establishment of physical borders and homotypic cell association, is critical for the establishment of distinct borders between adjacently developing organs. Failure in these processes could constitute the basis for pathological conditions such as organ fusion^2^,^3^,^49^.

## Discussion

Organ precursor cells need to be positioned in a configuration compatible with their interaction with other cell types and with the final organ shape and position in the body. Such interactions that maintain cells at a specific location are characteristic for organogenesis and differ from situations where arrival of cells at their target is followed by migration away after the completion of a certain task (for example, in the case of neutrophils arriving at the site of tissue damage^50^). To study this critical step in organ formation, we used the localization of PGCs during early gonadogenesis as a model. We focused on the step immediately following the chemokine-guided migration of PGCs, to study the mechanisms maintaining the cells at the target region, before stable interactions between germline and somatic gonad cells are established.

**Figure 8:**
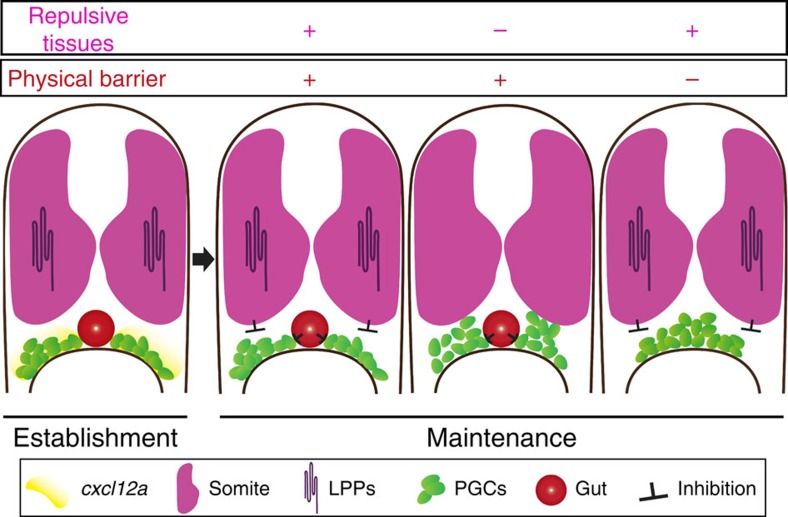
Progenitor cell positioning at target site. An illustration demonstrating the interplay of repulsive cues and physical barriers within the embryo that govern the positioning of PGCs at the site of the developing gonad. At the time of PGC clusters initial formation, the guidance cue *cxcl12a* RNA is expressed at the migration target (yellow). In the following stages the chemokine is not expressed, calling for other mechanisms maintaining the position of the germline progenitors. In wild-type embryos dorsal repulsive tissues (somites expressing LPPs in magenta) inhibit the dorsal migration of PGC clusters, while the developing gut (red) acts as a physical barrier separating the clusters, thereby contributing to the formation of distinct cell clusters at the site of developing organ. In embryos deficient for the function of LPPs or lacking the physical barrier, the PGCs exhibit abnormal positioning.

We found that after arriving at the region where the gonad develops, the positioning of the PGCs along the plane studied (transverse plane) is governed by physical and repulsive cues, rather than by chemokine signalling that guided the cells at earlier stages (Fig. 8). The chemokine receptor is known to undergo desensitization, internalization and degradation at domains of high-chemokine abundance^51^,^52^. Therefore, maintaining the cells within the region of the developing gonad for a long time by employing chemokine signalling would have required strong continuous production of the receptor and its ligand. Employing nearby developing tissues as physical barriers, combined with repulsive cellular domains, could serve this purpose more efficiently. Indeed, localized LPP overexpression converted practically any region in the embryo into a repulsive domain for PGCs. This observation indicates that the substrates for the LPPs are present throughout the embryo during early development, a period at which the relevant phospholipids could be searched for. This finding together with the fact that other developing organs could serve as physical barriers for other migratory cell populations, makes it likely that the two activities described in this work operate in patterning of other organs as well.

Barrier tissues have been implicated in a broad range of biological processes^53^,^54^,^55^. Following contact with the gut, PGCs undergo polarity changes resulting in an alteration in the direction of migration, demonstrating for the first time the precise response of cells interacting with such barriers *in vivo*. Consequently, the PGCs migrate away from the border to the region where the gonad develops, thereby maintaining two bilaterally positioned cell clusters. On the basis of the following evidence, we favour the idea that the gut constitutes a physical barrier for the migrating PGCs. First, the migration away from the barrier involved physical interaction between the migrating cells and the gut, arguing against the action of long-range repulsive cues. Second, 30% of the cell behaviours were characterized by prolonged contact time with the barrier, a behaviour distinct from that described for contact-mediated repulsion in the context of growth cone and cell migration (for example, in the case of Eph/ephrin function where an immediate response is observed^56^) and the fact that expression of putative repellent molecules in the gut such as Robo/Slit^57^ or ephrin^58^ was not described.

An interesting future investigation path is the exploration of the inherent properties of the cells comprising barrier tissues and of the mechanisms regulating the polarity change of migratory cells following interaction with such cellular entities. Similarly, it would be interesting to understand the cellular response of PGCs to repulsive signals and the mechanisms by which such signals counteract the attractive cues provided by chemokines. In addition, the development and localization of somatic gonadal cells in zebrafish and the mechanisms facilitating their interaction with the PGC clusters and the effects one population exerts on the other are of significant interest^15^,^16^,^59^.

The observation that PGCs migrate away from each other following contact (for example, the last section in the Supplementary Movie 8) despite the extended interaction time among the cells at this stage, is reminiscent of a behaviour termed contact inhibition of locomotion (protrusion collapse at the contact site and migration in the opposite direction following repolarization^46^,^60^). At high cell concentrations, continuous collapse of such protrusions, coupled to proper adhesion level, could contribute to the formation of the PGC clusters at this stage. Indeed, based on the simulations, adhesion among the PGCs seem to be essential for the formation of cell clusters that exhibit the same properties as those observed *in vivo* concerning size and positioning within the channel. Importantly, we find that PGCs change their polarity and migrate away from a physical barrier following the interaction with it. Interestingly, in such cases we observed a dramatic change in cell polarity manifested by elevation of actin polymerization on the opposite side of the cell, before the actual change in morphological polarity and migration in the other direction (Supplementary Movie 6). This suggests that the interaction with cells of the physical barrier is followed by the activation of a signalling cascade. The events discussed above could contribute to the reduction in PGC migration speed following arrival at the gonad region, which could in principle promote the formation of more stable interaction between PGCs and somatic cells of the developing gonad.

Defects in organogenesis involving organ fusion events have been reported, but the developmental and cellular basis for these abnormalities is unknown. For example, a fusion of the testicles that was associated with azoospermia and infertility has been reported in humans^61^ and the fusion of ovaries has been observed in sheep^62^. Pathological fusion of normally separated internal organs has also been reported in humans. In this case, fusion of testicles with the tissues of endodermal origin such as the spleen^2^ and the liver^3^ results in infertility. Thus, determining the molecular pathways controlling the maintenance of separated organs can contribute to the understanding of clinical conditions.

Finally, our findings concerning the localization of cells by a combination of physical barrier formation, repulsive cues and preferential interaction among group members are relevant at other scales as well. Natural and man-made barriers and prey–predator interactions were analogously shown to play a role in determining the migration and the distribution pattern of animal herds and ant colonies^63^,^64^,^65^.

## Methods

### Zebrafish strains and maintenance

A list of zebrafish lines used in this work is provided in the Supplementary Table 7. To prevent the pigmentation in embryos older than 24 hpf, 0.003% 1-phenyl-2-thiourea (P7629, Sigma-Aldrich) in 0.3 × Danieau's solution (17.4 mM NaCl, 0.21 mM KCl, 0.12 mM MgSO_4_·7H_2_O, 0.18 mM Ca(NO_3_)_2_, 1.5 mM Hepes) was used. The zebrafish were handled according to the regulations of the state of North Rhine-Westphalia, supervised by the veterinarian office of the city of Muenster.

### RNA expression constructs, morpholinos and microinjections

Capped sense mRNAs were synthesized using the mMessage mMachine kit (Ambion). RNAs and/or MO (Genetools, OR) were injected into the yolk of one-cell stage embryos unless stated otherwise. To assess the functionality of the translation-blocking MO antisense oligonucleotides, reporters containing the MO antisense oligonucleotide-binding site cloned upstream of the open reading frame of the gene of interest, followed by the *egfp* coding sequence were used. The functionality of the splice-blocking *lpp1* MO antisense oligonucleotide was verified by a PCR-based assay. A description of the constructs, primers and MO used is provided in Supplementary Tables 8,9 and 4, respectively.

### The RNAscope procedure and *in situ* hybridization

Whole-mount *in situ* hybridization was performed according to ref. 30, with the detailed protocol provided in Supplementary Method 1. Digoxigenin and fluorescein-labelled probes for chromogenic *in situ* were synthesized according to the manufacturer's protocol (Roche, Switzerland). Images of Chromogenic WISH and histological sections were captured on an Axioplan2 microscope (Zeiss) using a SPOT Xplorer camera (SPOT Imaging Solutions) controlled by either Metamorph (Visitron Systems) or SPOT (SPOT Imaging Solutions) software and processed using ImageJ (NIH). RNAscope procedure was performed according to ref. 31, with the detailed protocol provided in Supplementary Method 2 and imaged as z-stacks of whole embryos using a Z.1 light-sheet microscope (Zeiss) controlled by ZEN software (Zeiss) followed by image processing using Imaris software (Bitplane, Switzerland; Fig. 3; Supplementary Fig. 7). A list of the probes and the primers used to amplify them is provided in Supplementary Tables 8–10.

### Transplantation experiments

Donor and host embryos were engineered to be depleted for or to express specific proteins as indicated in the figures. Cells from a 4 hpf donor embryo were transplanted into a 4 hpf host using an Eppendorf CellTram Vario, and cell behaviour was immediately followed by time-lapse movies using an Epifluorescence microscope (Axioplan2, Zeiss).

### CRISPR/Cas9-based experiments

A sgRNAs set targeting the six *lpp* genes and one control set targeting the *albino*, *tyrosinase* and *golden* (each gene targeted by three to five sgRNAs^37^) were designed using CHOPCHOP^66^ (Supplementary Table 3). The templates for sgRNAs contain Sp6 promoter sequence (5′-ATTTAGGTGACACTATA-3′), 5′-GA sequence as the efficient consensus initiation site^67^, 18-base gene-specific site without the PAM (Supplementary Table 3—underlined sequences) and 23-base complementary region to a constant oligonucleotide encoding the reverse-complement of the trans-activating CRISPR RNA (tracrRNA) tail^68^. The single-stranded DNA overhangs were filled using DNA polymerase, Klenow fragment (Thermo Fischer Scientific) and the product is purified using QIAquick PCR purification kit (Qiagen). The sgRNAs were synthesized using MEGAscript Sp6 transcription kit (Ambion). The experimental and control sgRNAs sets were mixed with Cas9 protein and injected into the cell of one-cell stage embryos. At 28 hpf live embryos were imaged (Supplementary Figs 4a and 5a) or fixed for analysis using the RNAscope procedure before imaging (Fig. 3a). Z-stacks of embryos were imaged on a Z.1 light-sheet microscope (Zeiss), analysed and processed employing median filter, background subtraction and the ‘surface' module of the Imaris software to further reduce background originating in the notochord (Bitplane, Switzerland). For molecular detection of the mutations, one *lpp* sgRNA was coinjected with Cas9 protein, with embryos injected with Cas9 protein and a single *tyr* sgRNA (G416 in Supplementary Table 3) serving as controls. Genomic DNA was extracted from single treated embryos and the sgRNA target region amplified and sequenced.

### RNA-seq-based gene expression analysis

PGCs from 7 and 36 hpf embryos carrying the *kop-egfp-f-nos3′* transgene were sorted using FACS (FACSAria cell sorter (BD Biosciences) equipped with a 70 μm nozzle). 500 ng of total RNA from PGCs and somatic cells was isolated using the PicoPure RNA extraction kit (Arcturus; Alphametrix). Generation of the RNA-seq library and Illumina-based high-throughput sequencing were performed in the Microarray Hybridization and Analysis Core of the Johns Hopkins University (USA). 7 hpf samples were sequenced on GAII platform, with ∼25 million paired reads per sample (2X75); 36 hpf samples were sequenced on HiSeq 2000 platform, with 150 million paired reads per sample (2X100). The reads were filtered and trimmed for low-quality reads and then mapped to the transcriptome using SeqMap (Bowtie for the last two samples) and generated expression value using rSeq. Differential expression was detected using DEseq package of Bioconductor. None of the transcripts were excluded. The expression levels of LPA and S1P receptors, *sox32* RNA and cell adhesion molecules are presented in Supplementary Tables 2,5 and 6.

### Histological sectioning

Following the standard chromogenic *in situ* hybridization protocols (Fig. 2b) or fixation of embryos expressing fluorescent proteins (Fig. 5b), the samples were embedded in 4% low-melting agarose and sectioned into 100 μm slices using a vibratome (Leica, Germany). Overnight incubation in Hoechst 33342 (Life Technologies) was performed at 4 °C (1:10,000; Fig. 5b). The fluorescent sections were mounted on slides using fluorescence mounting medium (Dako, Germany) and imaged to generate z-stacks of 3-μm interval using an LSM 710 confocal microscope (Zeiss) followed by processing using ImageJ (NIH).

### Preparation for microscopy

Embryos were anaesthetised using 0.03% tricaine methasulfonate (tricaine A5040, Sigma-Aldrich) in either 0.3 × Danieau's solution or embryo medium before live imaging. For embryos older than 24 hpf, pigmentation was inhibited by the addition of 0.003% 1-phenyl-2-thiourea to the embryo medium at the end of gastrulation. Images and time-lapse movies were captured using water-immersion objectives. Detailed experimental setups are presented in Supplementary Table 1.

### Three-dimensional time-lapse microscopy

Two-photon laser scanning microscopy (Supplementary Movie 2) was performed using a Leica SP5 DM5000 upright microscope, a dipping lens objective (Olympus XLUMPFL 20 × 0.95 NA) and simultaneous excitation at 980 nm (Mai Tai Spectra Physics) and 1,030 nm (t-pulse 20 Amplitude Systems)^69^. The spatial resolution was 1.37 μm^3^ and the temporal resolution 2 min. Supplementary Movie 2 was generated using the Amira software (Visage Imaging, CA). Germ cell tracking was performed using ImageJ (NIH). Epifluorescence movies were captured on an Axioplan2 epifluorescence microscope (Zeiss). Image analysis was performed using ImageJ (NIH) and Metamorph (Visitron Systems) software packages. Light-sheet recordings of live zebrafish embryos (Supplementary Movies 1 and 5–8) were performed using SiMView microscopy^27^. The embryos were embedded at 24 hpf in 1% low-melting temperature agarose (Sigma-Aldrich, A4018) and recorded using either a 16 × objective and 1.5–3 min time intervals, or a 40 × objective and 30 s time intervals followed by analysis using Imaris software (Bitplane, Switzerland; Fig. 6d–f). When necessary, time-dependent intensity levels and the embryo position were corrected computationally using the Fiji distribution of ImageJ (plugins ‘Bleach Correction' and ‘StackReg', respectively) and custom software written in Matlab (The MathWorks)^70^.

### Statistical analysis

All statistical analysis was performed using PRISM (Graphpad Software, Inc.) and Microscoft Excel.

### Cluster formation analysis and simulations

To determine the distribution of PGCs at the gonad region, epifluorescence z-stack images were captured at 24, 24.5 and 25 hpf (Fig. 7c). Following z-projection, the positions of PGCs, the outline of the embryos and the gut were manually marked and measured using ImageJ and normalized using Microsoft Excel. For PGC behaviour analysis at the gonad region SiMView z-projected time-lapse movies were analysed (Fig. 6f; Supplementary Movies 1,5 and 7). Median filter and edge detection ImageJ plugins were employed on the movies. Absolute xy positions of PGCs were manually measured and marked using ImageJ and cell speed and contact time were calculated using Microsoft Excel.

The particle-based simulations were performed using the parameters derived from epifluorescence z-stacks or SiMView time-lapse movies in zebrafish embryos. We, therefore, used 11 μm as the cell diameter, 0.6 μm min^−1^ (0.05 cell diameter per min) as the cell velocity, 5 × 36 cell diameter as the gonad region area in two dimensions (since the third dimension is only two to three particle-diameter long), *D*_*r*_=1/60 min as the rate of change of direction of motion and 14 cells at the site of the gonad. We modelled the cells as self-propelled point-particles whose motion is determined by the overdamped equation:


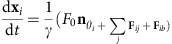


where **x**_*i*_ is the position of the *i*th particle and *γ* is the Stokes drag coefficient. The force acting on the *i*th particle is composed of the motility force of the particle and forces that other particles and boundaries exert on it:

**F**_*ib*_ is the force exerted on the *i*th particle by boundaries and **F**_*ij*_ is the force the *j*th particle exerts on the *i*th, derived from a Lennard-Jones potential:


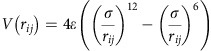


where *r*_*ij*_ is the relative position of particles *i* and *j*. *ɛ* is the depth of the potential well and represents cell–cell adhesion strength. Note that for *ɛ*=0 the potential vanishes and the particles do not interact.

*F*_0_ is the magnitude of the self-propulsion force. The unit vector _*i*_ denotes the direction of the propulsion force. The angle of its direction with respect to the *x* axis diffuses:


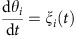


Where *ξ*_*i*_ is Gaussian white noise which satisfies:


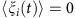



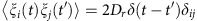


and *D*_*r*_ is the rotational diffusion rate.

We numerically integrated the Langevin equations of motion using the Euler method, considering the gonad region to be a two-dimensional area. We employed periodic boundary conditions along the long axis of the box (dashed lines in Supplementary Movies 9–10) and hard walls (implemented by steep repulsive potentials, used here to exert a repulsive force and does not implicate a production of repulsive conditions in the biological meaning) on the other axis (solid black lines in Supplementary Movies 9–10). The periodic conditions at the top and bottom of the channel were employed for maintaining the number of cells in the chamber (as is the situation *in vivo*) and since the actual properties of these boundaries are not known. In the ‘reflective boundary' model (Fig. 7a; Supplementary Movies 10), in addition to the repulsive potential, when a cell touches the boundaries (meaning that it is closer than a small threshold distance), the direction of its self-propulsion force is immediately changed perpendicular to the wall.

## Additional information

**Accession codes:** The RNA-seq data have been submitted to the NCBI Gene Expression Omnibus (GEO) with the accession number GSE77077 .

**How to cite this article:** Paksa, A. *et al.* Repulsive cues combined with physical barriers and cell–cell adhesion determine progenitor cell positioning during organogenesis. *Nat. Commun.* 7:11288 doi: 10.1038/ncomms11288 (2016).

## Supplementary Material

Supplementary Information Supplementary Figures 1-15, Supplementary Tables 1-10, Supplementary Note 1, Supplementary Methods 1- 2 and Supplementary References.

Supplementary Movie 1 PGC clusters located deep within the tissue imaged in transgenic embryos whose PGCs are labeled by Tg(kop:egfp-f-3'nos3) and the developing gut is by Tg(sox17:dsred) using SiMView light-sheet microscopy. Strong active motility of PGCs in separate clusters is observed following arrival at the target (24-27hpf in first movie) and 10 hours later (31-34hpf in the second movie). Movies optimized for contrast and processed for edge detection using ImageJ. Scale bars 25μm.

Supplementary Movie 2 A two-photon movie showing PGC behavior in a cluster. Lateral view of a transgenic Tg(kop:egfp-f-3'nos3) zebrafish embryo whose PGC membranes are shown in turquoise and somatic cell nuclei labeled in red by injection of h2b-mcherry RNA. The green track of a PGC indicates anterior-posterior migration, whereas the yellow track lateral-medial migration of the same cell. Scale bar 25μm. Anterior is to the left and dorsal is up. See also Fig. 1c.

Supplementary Movie 3 Cells overexpressing Cxcl12a together with either a photo-activatable GFP (control) or LPP proteins, were transplanted into embryos lacking Cxcl12a (medny054), whose PGCs were labeled by EGFP. PGCs (in green) directionally migrate toward the Cxcl12a expressing cells (shown in red) and remain associated with them. PGCs do not associate with LPPs-expressing cells (starred cells in red) and migrate away from such cells. The starting time point of the movies indicates time after transplantation. Scale bar 50μm. See also Fig. 4d-e and Supplementary Fig. 9.

Supplementary Movie 4 PGCs, labeled by EGFP and visualized by epifluorescence microscope starting at 24.5hpf, show active migration within two clusters in wild-type embryos and within one cluster in embryos lacking the gut. Scale bar 100μm. Dorsal view. Anterior is up. See also Fig. 6a.

Supplementary Movie 5 PGCs (EGFP-marked) change their migration path upon contacting the gut tube (DsRedlabeled) as imaged using SiMView light-sheet microscopy starting at 26hpf. Cells were tracked using ImageJ. Scale bar 25μm. Dorsal view and anterior is up. See also Fig. 6b.

Supplementary Movie 6 High magnification view of a germ cell expressing Lifeact-Ruby protein labeling its actin structures at the cell front (presented in green) and farnesylated EGFP labeling the Golgi apparatus at the cell back (presented in red), as it interacts with the developing gut (labeled by a sox17:egfp transgene, presented in red). A SiMView light-sheet microscope was used to image a 25hpf embryo. Actin structures form at the side and the back of the PGC following its contact with the physical barrier and migration away from it. White arrows indicate the polarity of the cell. Scale bar 5μm. Dorsal view, anterior up. See also Fig. 6c.

Supplementary Movie 7 A movie similar to Supplementary Movie 6, where the migrating cell (asterisk) is surrounded by other PGCs in a 25.5hpf embryo. The initial migration direction of the PGC is depicted by an arrow at time point 0. The circles designate the contact of the PGC with either the gut or another PGC. The cell undergoes continuous polarity rearrangements following contacting the gut or other PGCs, delaying establishment of stable polarity (new front) allowing migration away from the gut and other PGCs. Scale bar 10μm. Dorsal view, anterior is up.

Supplementary Movie 8 A PGC (starred, red cell) without interactions with the gut (green) or other PGCs, followed by a presentation of another PGC touching the gut tube (PGC-gut contact). The last section of the movie presents a representative case of a PGC interacting for a prolonged time with another PGC at the gonad region (PGC-PGC contact). Scale bars 25μm. Movies captured at 26hpf.

Supplementary Movie 9 Simulation of PGC distribution and cell cluster formation at the gonad region employing non-reflective boundaries (solid black lines). The dashed lines indicate periodic boundaries. The movie presents different adhesion levels (0.1 to 0.3). The two dimensional area of the gonad region is defined based on in vivo measurements (5 x 36 in cell diameter). t designates time in min. The simulations have been performed starting at t=0, but the steady-state is presented ranging from 4000-5000min.

Supplementary Movie 10 Similar to Supplementary Movie 9, implementing reflective boundaries at the gonad region (solid black lines).
