# Supplementary material for: Repulsive cues combined with physical barriers and cell–cell adhesion determine progenitor cell positioning during organogenesis
Source: Nat Commun. 2016 Apr 18;7:11288. doi: 10.1038/ncomms11288 (PMC4837475; doi:10.1038/ncomms11288)
Supplement: Supplementary Information — Supplementary Figures 1-15, Supplementary Tables 1-10, Supplementary Note 1, Supplementary Methods 1- 2 and Supplementary References. [file ncomms11288-s1.pdf]

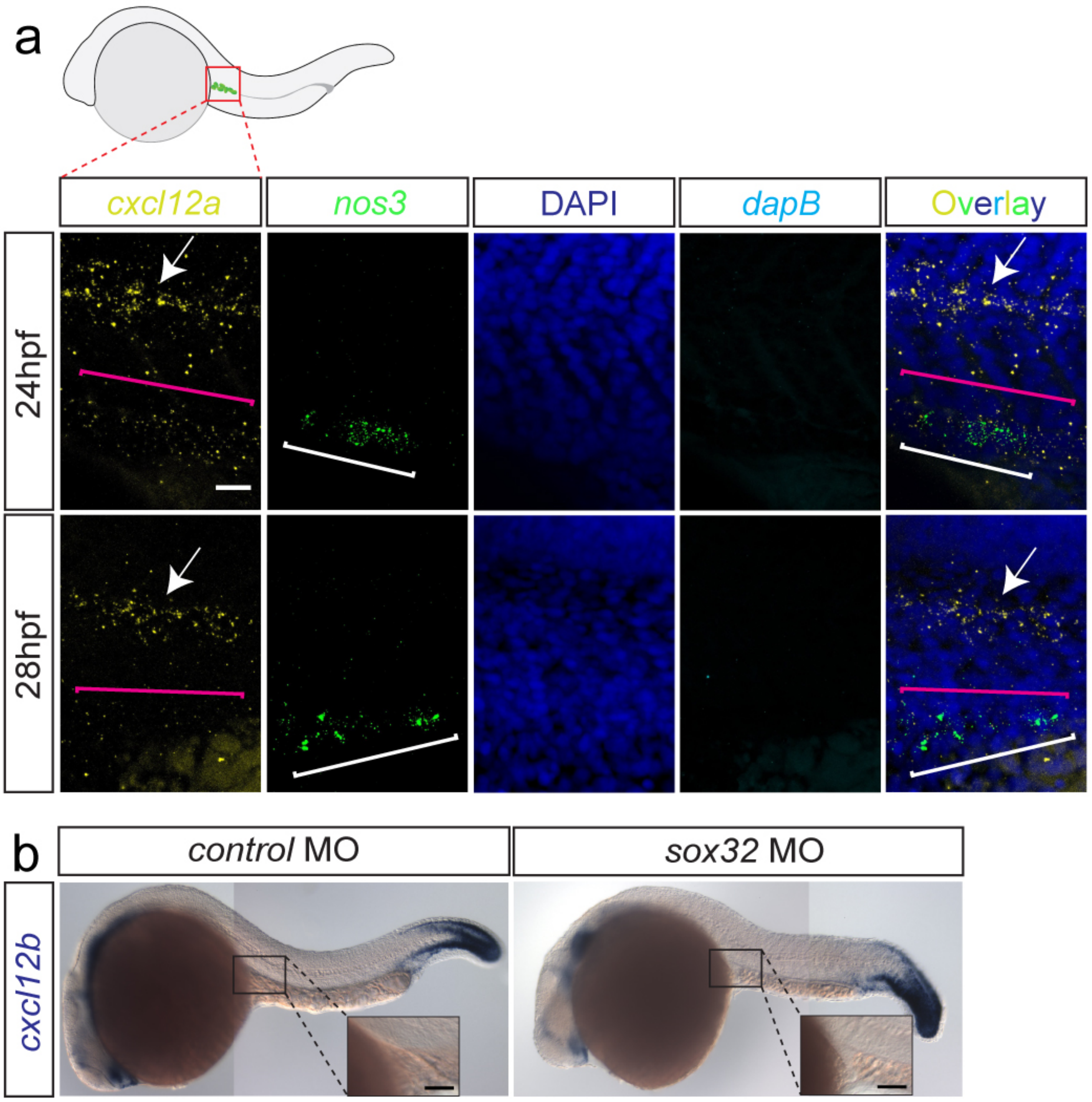

Supplementary Figure 1. *cxcl12a* and *cxcl12b* expression patterns following PGC arrival at the site where the gonad develops

(**a**) *cxcl12a* expression at the gonad region of 24hpf-embryos is detected (using the sensitive RNAscope protocol, magenta bracket in the upper panel), along with much higher expression level in the lateral line (arrow). At 28hpf, only weak background level *cxcl12a* is detected where the PGCs reside. PGCs detected based on *nanos3* (*nos3*) expression (white brackets). Nuclei stained with DAPI. *dapB* serves as a negative control. Merged overlay images presented on the right. Scale bar 25µm. Dorsal up. (**b**) *cxcl12b* RNA expression is not detectable at the gonad region in 24hpf control embryos, nor in embryos lacking the gut tube. Insets display a higher magnification of the gonad region. Scale bars 50µm.

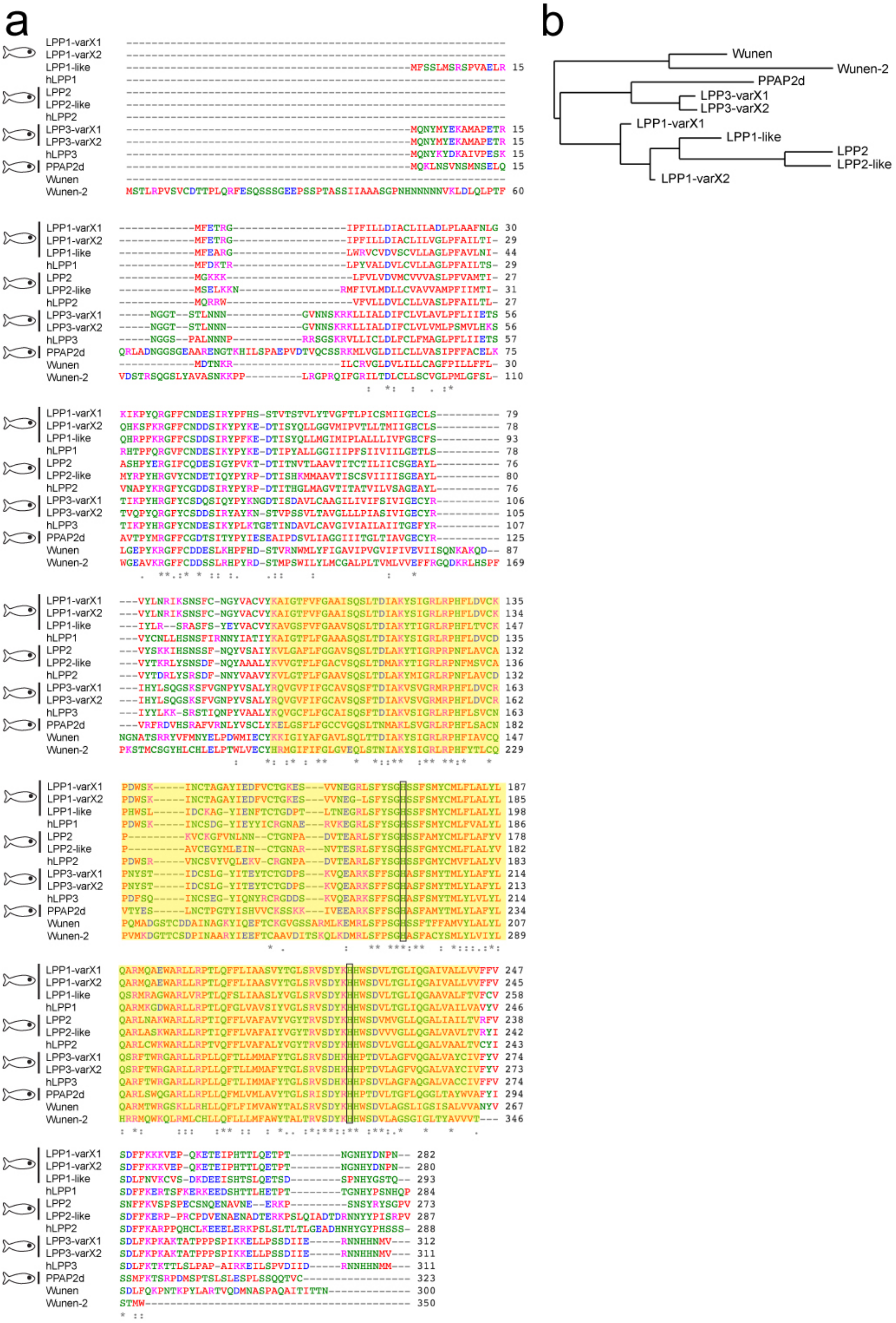
a
LPP1-varX1
LPP1-varX2
LPP1-like
hLPP1
LPP2
LPP2-like
hLPP2
LPP3-varX1
LPP3-varX2
hLPP3
PPAP2d
Wunen
Wunen-2
b
Wunen
Wunen-2
PPAP2d
LPP3-varX1
LPP3-varX2
LPP1-varX1
LPP1-like
LPP2
LPP2-like
LPP1-varX2

Supplementary Figure 2. Protein sequence alignment of Lipid Phosphate Phosphatase variants

(**a**) Zebrafish LPPs or PPAP2s and their splice variants are aligned with *Drosophila* Wunens and human LPPs (hLPPs). Phosphatase domain highlighted in yellow. Accession numbers as follows: Wunen (FBpp0087636), Wunen-2 (FBpp0087590), hLPP1 (AB000888.1), hLPP2 (AF047760.1) and hLPP3 (AB000889.1) and those of zebrafish LPP proteins provided in Supplementary Table 8. Two boxes display residues important for the catalytic activity (See Fig. 4a-c). LPP1-varX1, LPP1-varX2 and LPP3-varX1 exhibit the highest identity to the Drosophila Wunen protein (47%, 44% and 42% respectively) and to Wunen-2 protein (41%, 40% and 40% respectively). Identical amino acids among all sequences labeled by asterisks (*), conserved substitutions marked with colons (:) and semi-conserved substitutions indicated by periods (.). Red residues: Small and hydrophobic (including aromatic) amino acids; Blue residues: Acidic amino acids; Magenta: Basic; Green: Hydroxyl and sulfhydryl and amine; Grey: Unusual amino/imino acids. Alignment performed using ClustalW2 (EMBL-EBI). (**b**) Phylogenetic tree of *Drosophila* Wunens and zebrafish LPPs using online phylogeny.lirmm.fr tool[1].

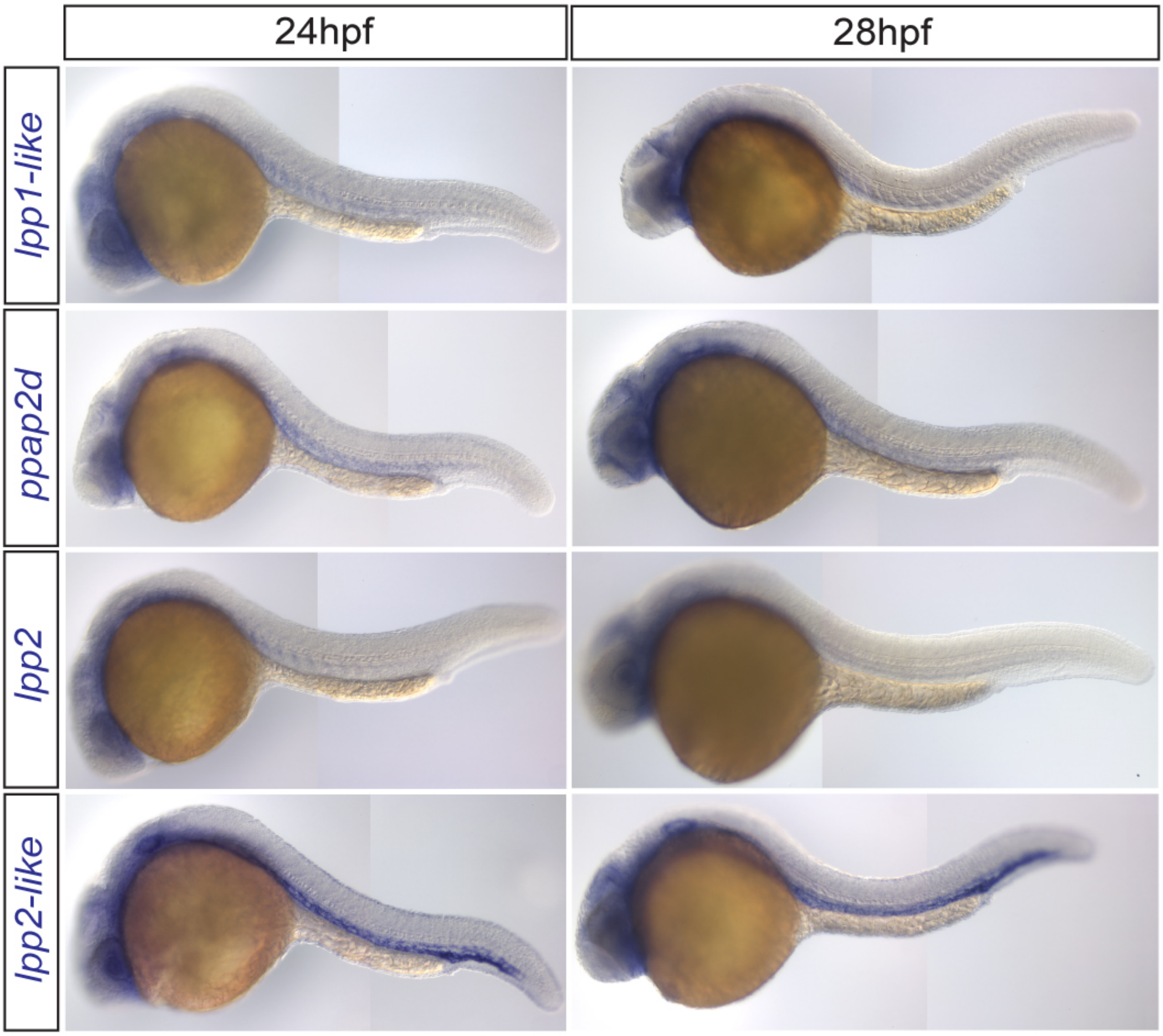

Supplementary Figure 3. RNA expression pattern of zebrafish *lpp* variants *lpp1-like*, *ppap2d* and *lpp2*

RNA expression pattern of the *lpp* indicated variants. See also Fig. 2b.

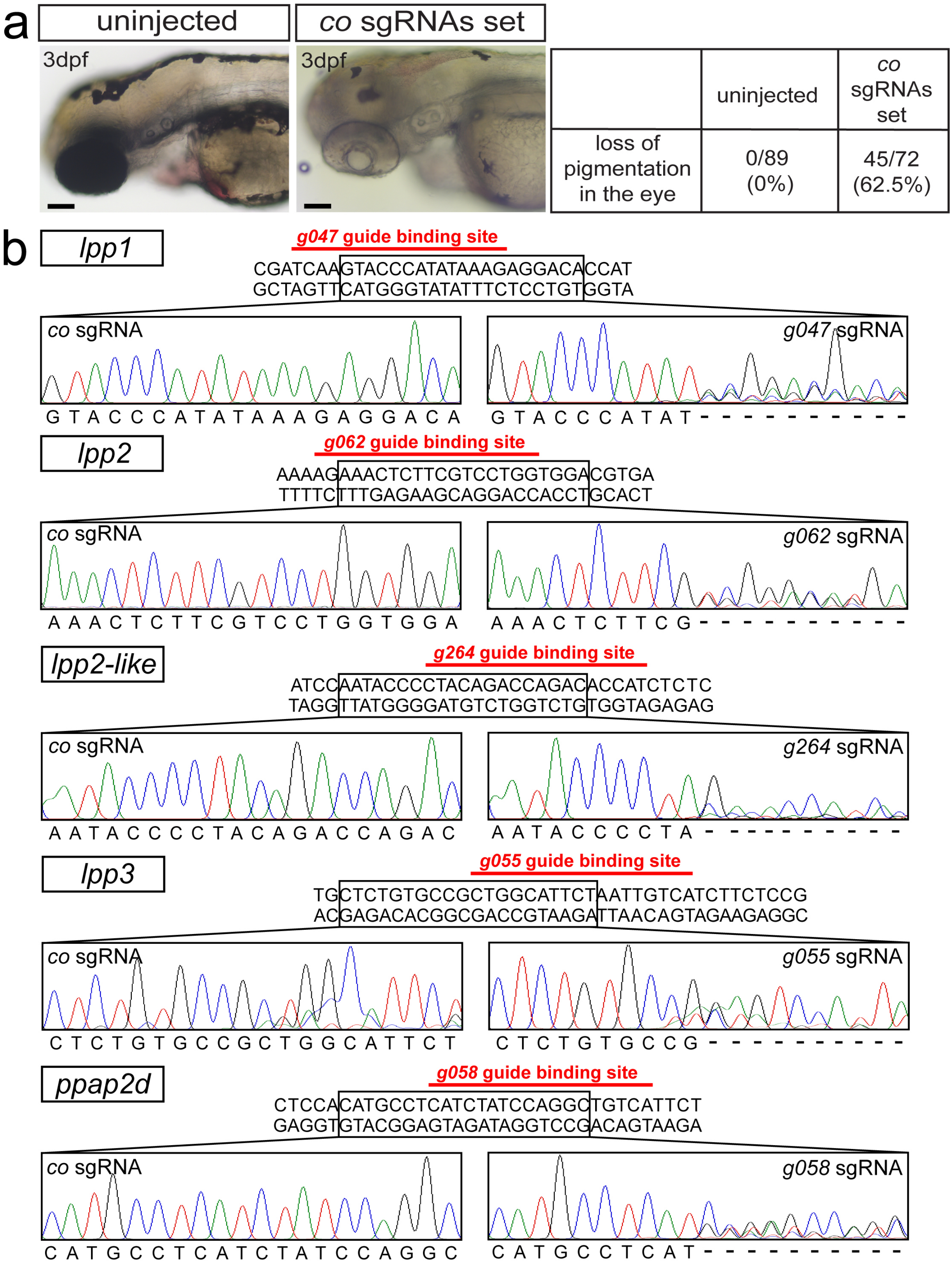

Supplementary Figure 4. Single guide RNAs efficiency in generating mutations
(**a**) Functionality of a set of *control* sgRNAs, demonstrated by loss of pigmentation in majority of the treated embryos at 3dpf as compared with uninjected embryos. Scale bars 200µm. (**b**) Efficiency of sgRNAs designed for *lpp* genes. Generation of the mutations by one sgRNA per gene-specific set (right panels), as evaluated by sequencing, compared with the results obtained using a *control* sgRNA (left panels) targeting the *tyrosinase* gene (G416 in Supplementary Table 3). Ambiguous sequence at the *lpp* sgRNA-binding region in the sequencing results confirms the efficiency of the genome-editing procedure.

a

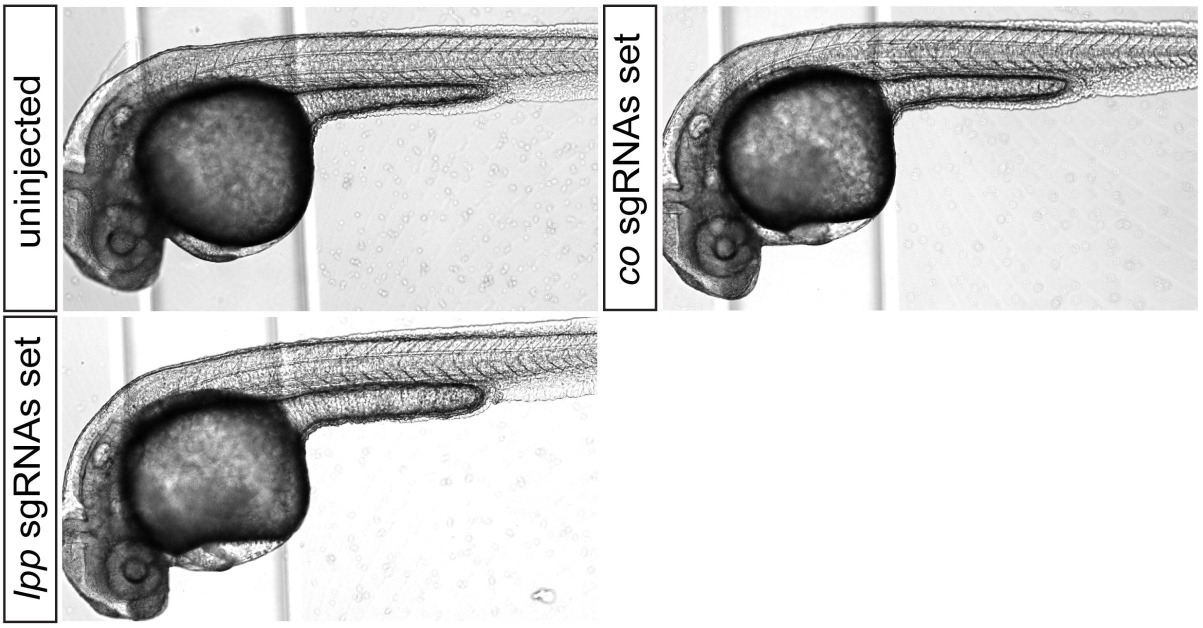

b

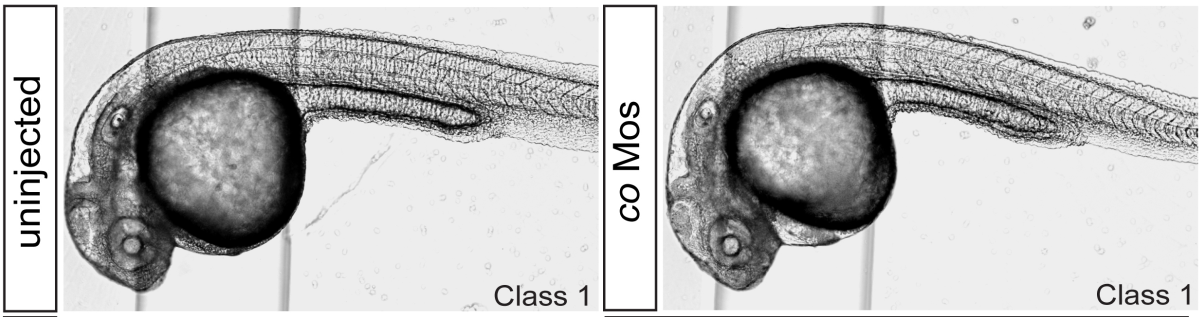

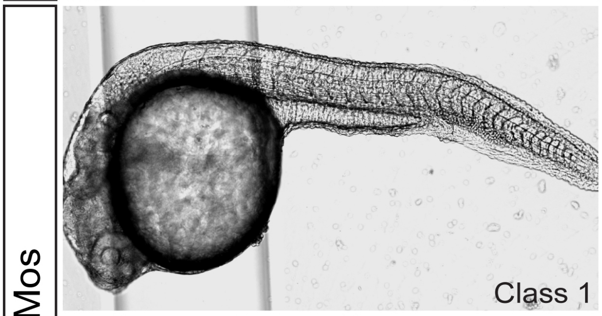

	uninjected	*co* MOs	*lpp* MOs
Class 1	324/356 (91%)	75/96 (78.1%)	339/395 (85.8%)
Class 2	0/356 (0%)	5/96 (5.2%)	10/395 (2.5%)
Class 3	8/356 (2.3%)	6/96 (6.3%)	11/395 (2.8%)
Class 4	24/356 (6.7%)	10/96 (10.4%)	35/395 (8.9%)

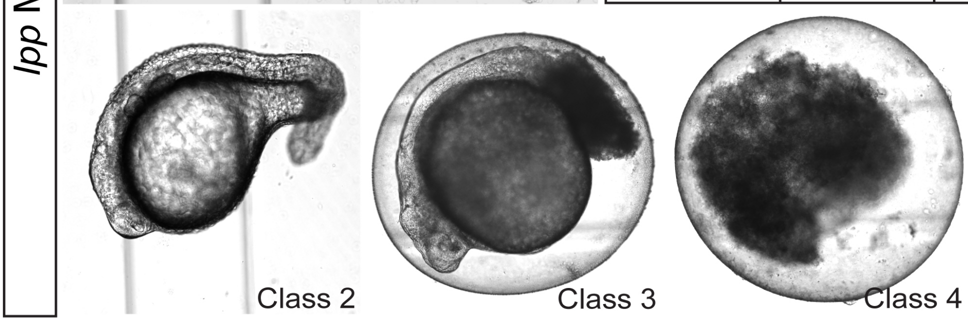

Supplementary Figure 5. Somatic phenotype in *lpp*-sgRNA and MO-treated embryos (**a**) 28hpf embryos showing no morphological defects when treated with a mixture of *lpp* sgRNAs (Number of embryos examined: N=340), similar to uninjected (N=460) and *control* sgRNAs-injected (N=280) embryos. (**b**) Images and data presenting a range of somatic phenotypes of MO-treated 28hpf embryos knocked down for *lpp* function, compared with uninjected and embryos treated with 6 *control* MOs. The class1 embryos, that are the least affected morphologically, were used to evaluate the role of LPPs in controlling PGC positioning.

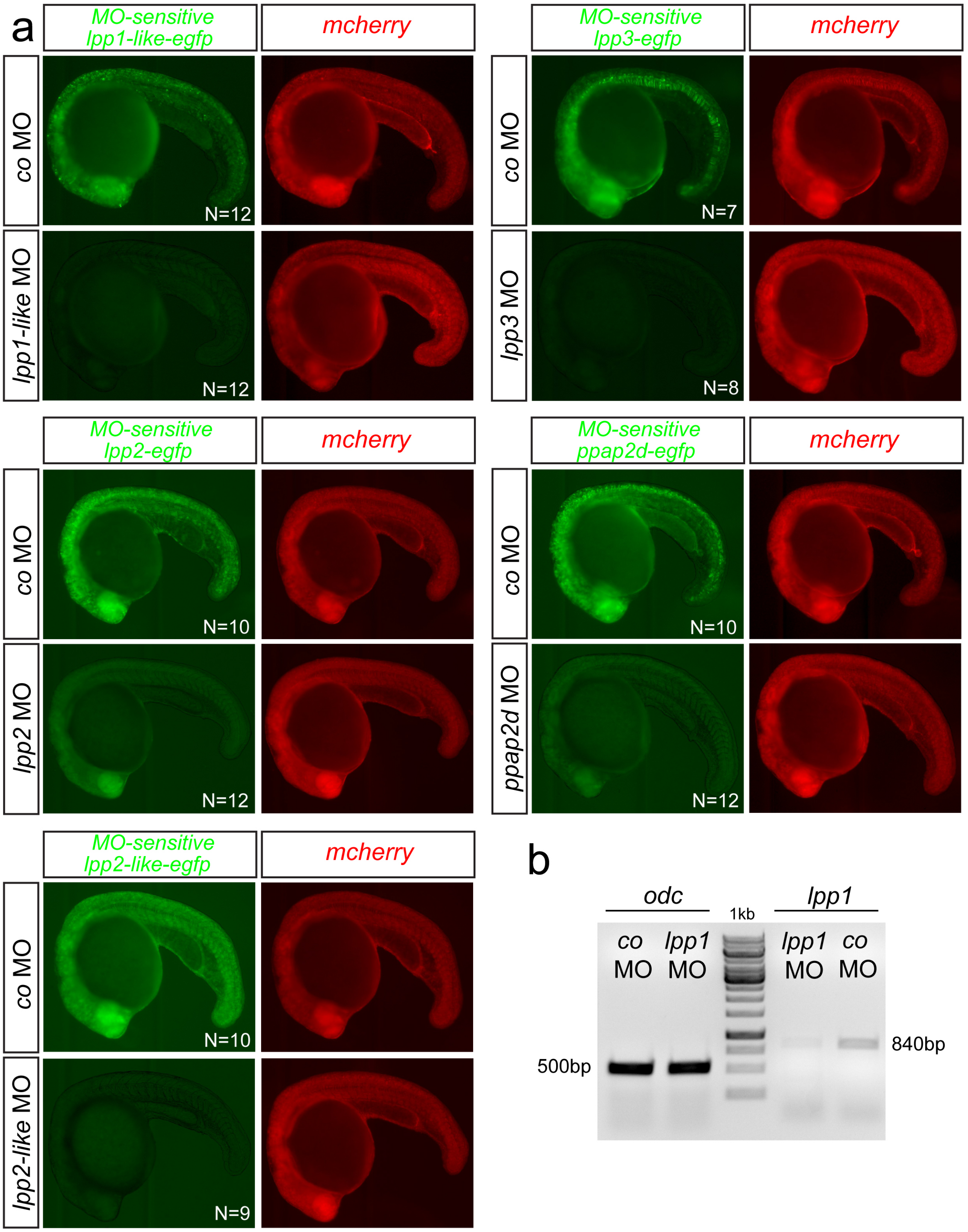

Supplementary Figure 6. Functionality of the *lpp* MOs

(**a**) Determination of the efficiency of translation-blocking MOs using EGFP reporters. *lpp* MOs specifically inhibit the translation of the downstream *egfp*, as demonstrated by the loss of EGFP signal, while the signal persists in *co* MO-treated embryos. *mcherry* is used as an unaffected control. N is the number of embryos assayed. (**b**) Functionality of *lpp1* splice-blocking MO using a PCR-based assay with primers presented in Supplementary Table 8. The mRNA level is largely reduced when employing the *lpp1* MO, as compared with *odc* used as control mRNA that is not affected. DNA marker: GeneRuler 1 kb DNA Ladder.

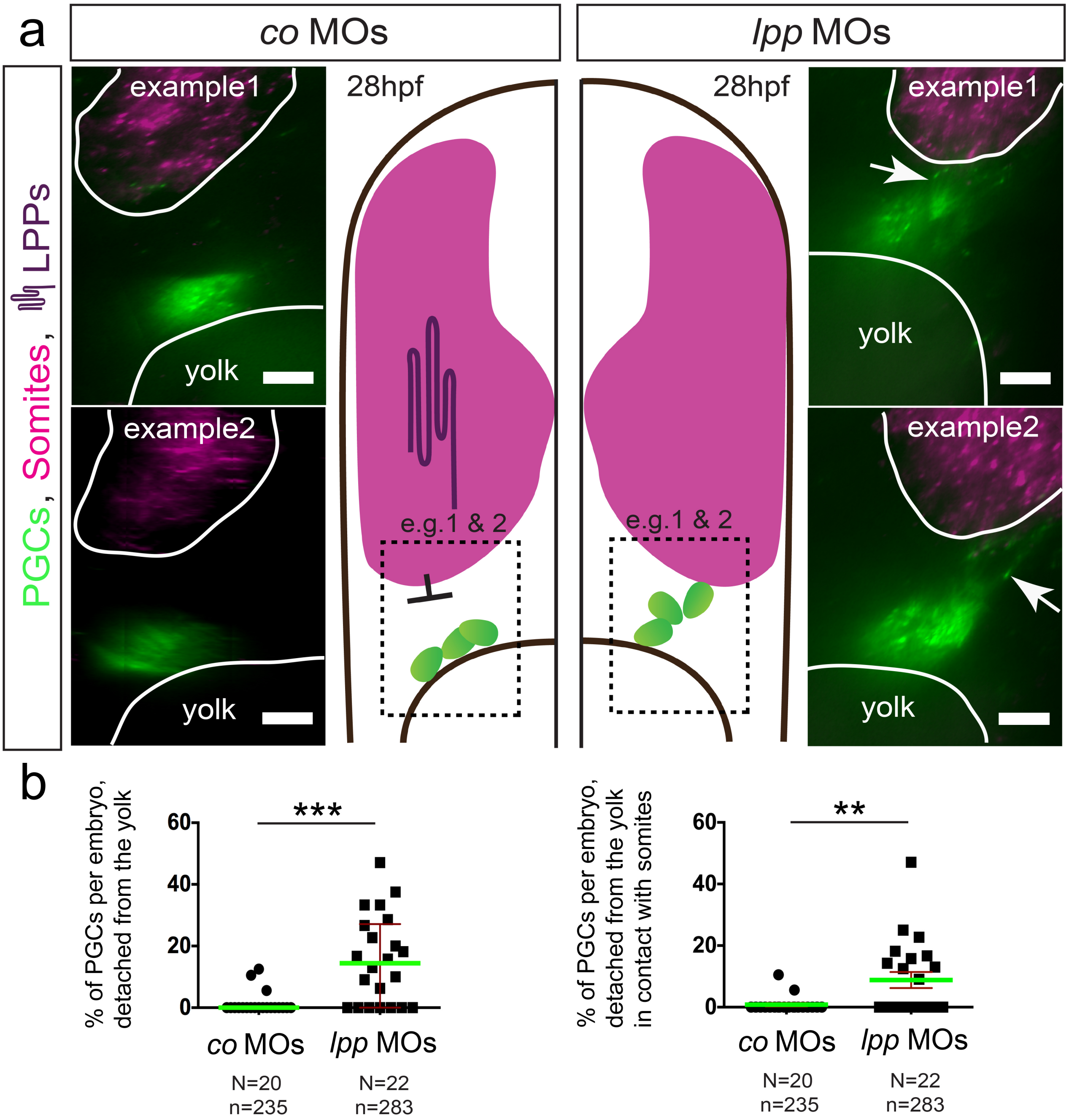

Supplementary Figure 7. PGCs contact the somites in embryos treated with MOs against *lpps*

(**a**) PGCs are labeled with EGFP (*Tg(kop:egfp-f-3'nos3)*) and *egfp* RNA, both shown in green, with the somites labeled using *myoD* (shown in magenta) employing the RNAscope procedure. PGCs contact the somites in LPP-knocked down embryos (right, examples 1 and 2), as compared with control embryos, in which PGCs are located at a distance from the somite border and in contact with the yolk (left, examples 1 and 2). (**b**) The percentage of PGCs per embryo that detached from the yolk (left) as well as those contacted the somites (right) in 28hpf embryos. Green lines represent the median and mean values and error bars indicate median with interquartile range and s.e.m. (left and right respectively). The statistical significance was evaluated using the Mann-Whitney U test (**$p \leq 0.01$ and ***$p \leq 0.001$). N is the number of embryos and n the number of PGCs examined. Scale bars signify 10μm. Dorsal is up.

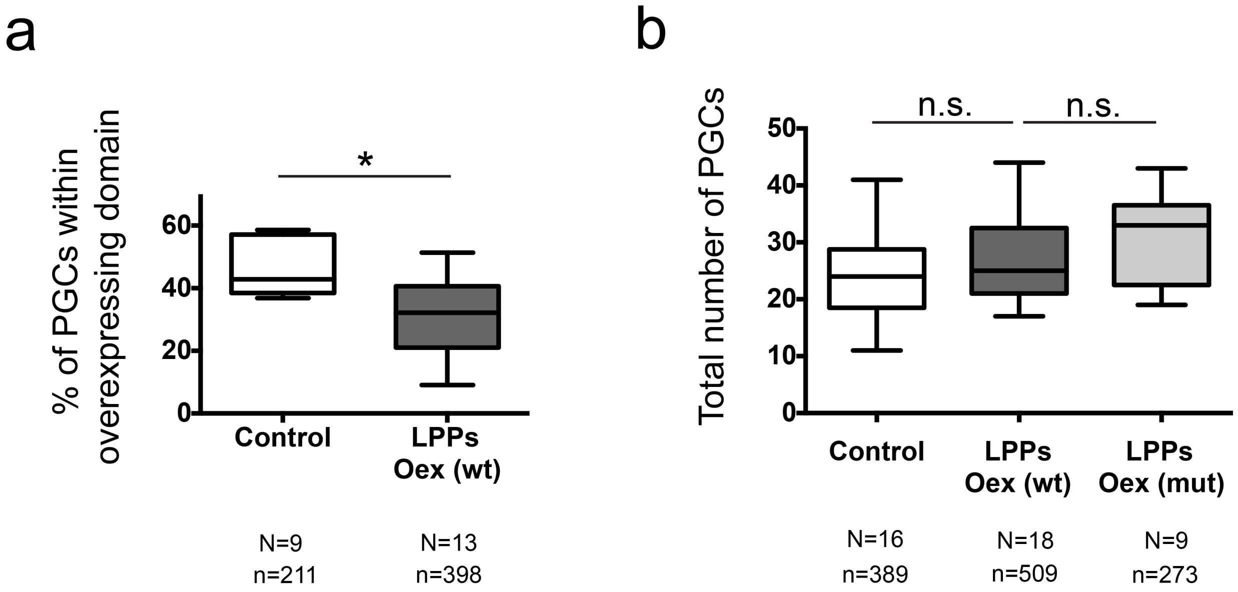

Supplementary Figure 8. PGCs vacate cellular domains where LPPs are overexpressed, but do not die within them

(**a**) A boxplot presenting a significantly reduced presence of PGCs within LPP-overexpressing domains in embryos lacking the function of Cxcl12a and Cxcl12b as compared with their distribution within control domains. Mann-Whitney U test, $*p \leq 0.05$. For experimental setup see Fig. 4a. (**b**) The total number of PGCs corresponding to Fig. 4a-c is not affected by exposure to LPP activity as determined by One-way Anova. Median values for control provided by black lines inside the boxes, error bars signify minimum to maximum range of the data points. N and n represent the number of embryos and PGCs respectively.

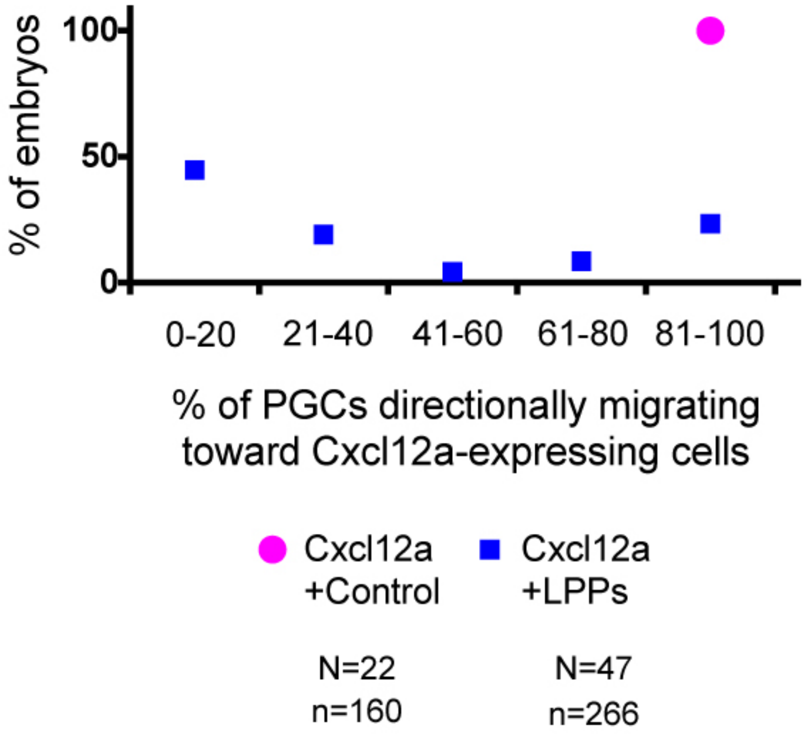

Supplementary Figure 9. LPPs interfere with Cxcl12a-mediated attractive activity.

Percentage of PGCs migrating toward the cells expressing Cxcl12a is reduced by co-expression of LPPs (1-varX1 + 3-varX1). Mann-Whitney U test, ****$p \leq 0.0001$. See also Fig. 4d-e and Supplementary Movie 3.

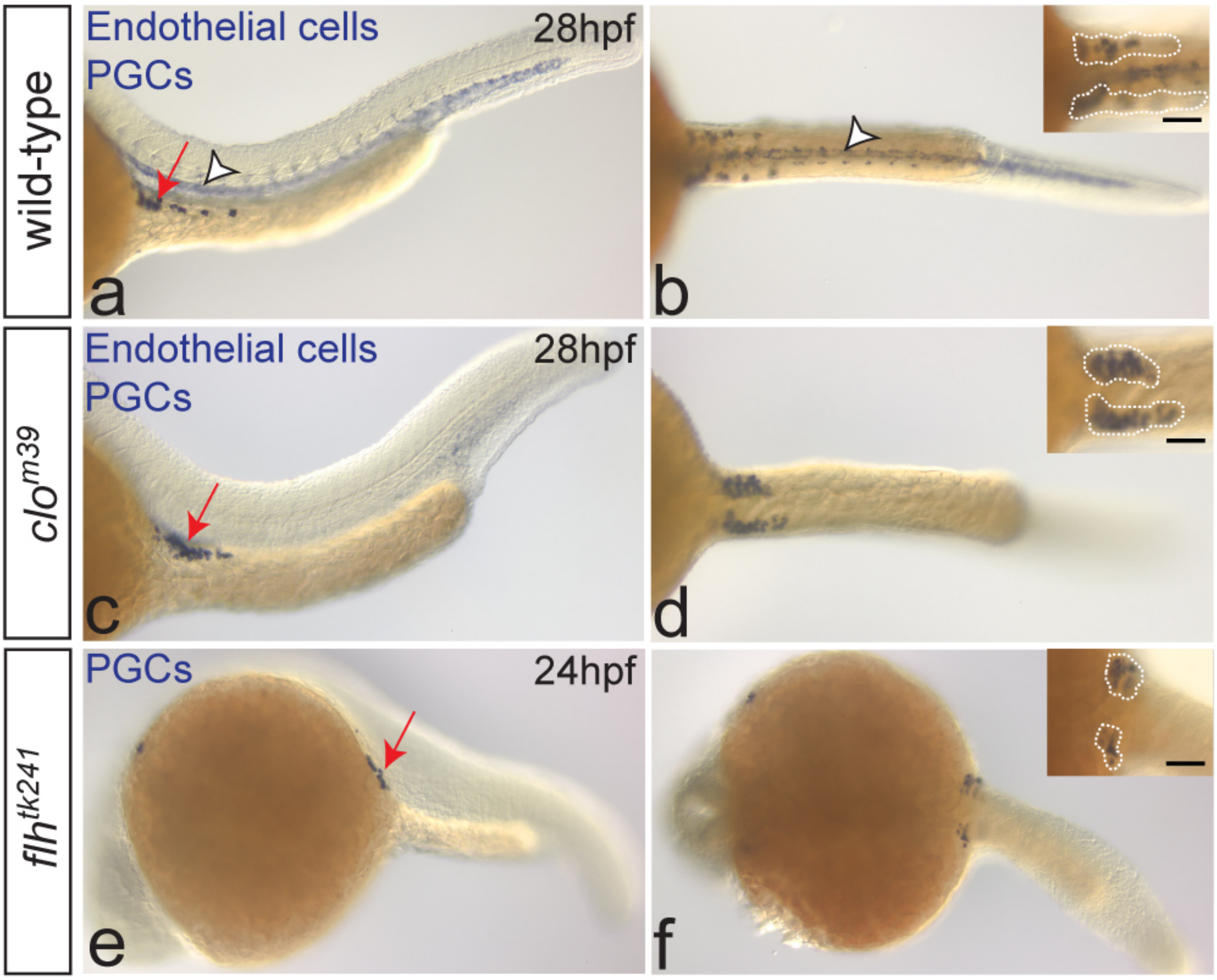

Supplementary Figure 10. Bilateral positioning of PGC clusters does not require the notochord or blood vessels.

One-color *in situ* hybridization on wild-type (**a**-**b**), *clo*^{m39} (**a**-**d**) and *flh*^{tk241} (**e**-**f**) embryos. PGCs are stained with *nos3* (a-f, arrows on the left-panel images) and endothelial cells with *kdrl* probe (**a**-**d**, arrowheads in **a**-**b**). Despite the lack of endothelial cells, PGCs arrive at the target (**c**, arrow) and form two separate clusters at 28hpf (**d**; dotted lines in inset) in *clo* mutant embryos (number of embryos examined: N=12), similar to wild-type embryos (**a**-**b**; N=60). In embryos lacking notochord (*flh*^{tk241}), PGCs form two bilateral clusters at the gonad region at 24hpf (**e**-**f**; dotted lines in inset; N=15). Scale bars 50μm. Lateral (left panels) and dorsal (right panels) views are shown.

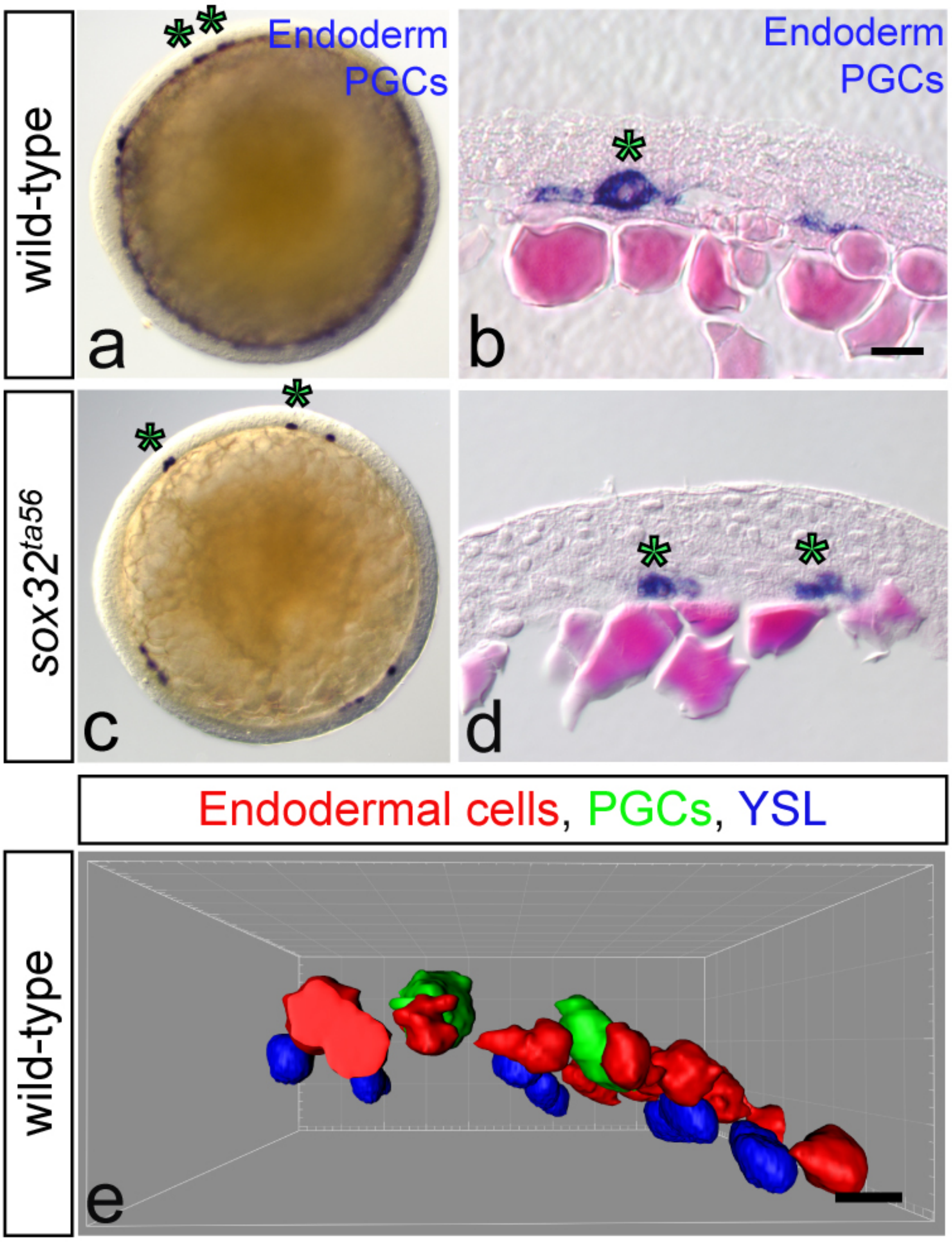

Supplementary Figure 11. PGC migration at early developmental stages does not require endoderm.

(**a**, **c**) Whole-mount *in situ* hybridization of *nos3* as a PGC marker (asterisks) and *sox17* as an endodermal marker (blue rim around the yolk in a), both stained in blue. Animal views are shown. (**b**, **d**) Hematoxylin and Eosin Counterstained histological sections of embryos following WISH using *nos3* (asterisks) and *sox17* (blue cells in b) probes. (**a**-**b**) Wild-type PGCs migrate in the vicinity of the yolk in close contact with endodermal cells. Number of embryos examined in **a**: N=20 and **b**: N=3. (**c**-**d**) In *sox32* mutant embryos lacking the endoderm PGCs migrate close to the yolk. **c**: N=20; **d**: N=10 (**e**) 3D Confocal Z-stacks of wild-type embryos in the lateral view. Embryos of transgenic fish with EGFP-labeled PGCs are injected with *secfp* RNA together with an activated *taram-A* transcript (*taram-A*)* into one blastomere at 32/ 64-cell stage for endodermal cell labeling, followed by YSL nuclei labeling with *h2b-mcherry* RNA upon injection into the yolk. PGCs (in green) are positioned among scattered endodermal cells (shown in red) and in the same layer with them, directly above the YSL nuclei (shown in blue) (N=4). Images were processed using Imaris. Scale bars 25µm. Embryos at 70-80% epiboly in all experiments.

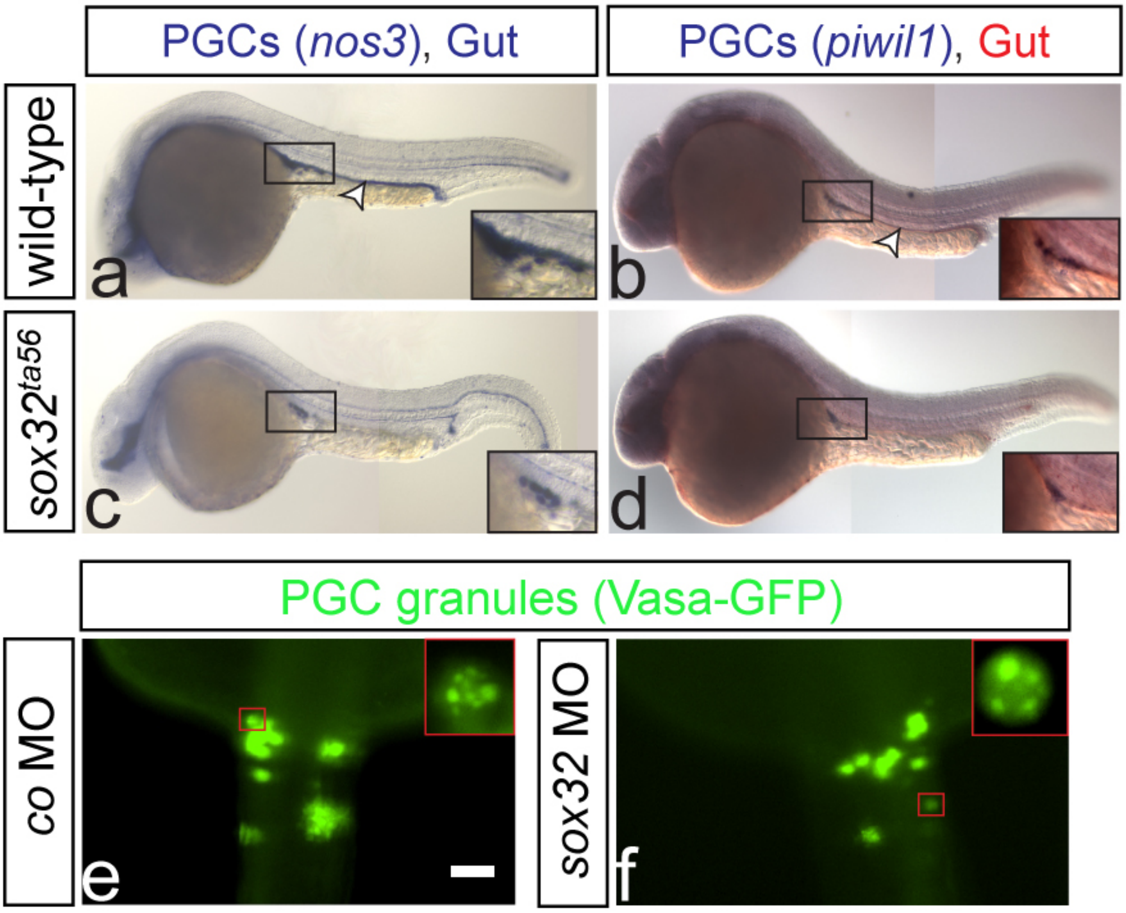

Supplementary Figure 12. PGCs are normally specified in embryos lacking the gut tube

One color (**a** and **c**) or two color (**b** and **d**) WISH on wild-type and *sox32*^{ta56} embryos at 24hpf (a and c respectively) or 28hpf (b and d respectively). The gut is stained using *foxa1* probe (a-d). In wild-type and *sox32* mutant embryos the PGCs (insets) express both the early marker *nos3* and the late marker *piwil1.* Arrowheads indicate the position of the gut (a-b), and no *foxa1* expression is detected in the mutants (c-d). Number of embryos examined in **a**: N=40, **b**: N=20, **c**: N=9 and **d**: N=5. (**e**-**f**) PGCs are labeled with *vasa-gfp* mRNA, showing the characteristic granule formation in PGCs (insets in e and f respectively). **e**: N=14 and **f**: N=15. Scale bar 50μm. Dorsal views are shown. Anterior is up.

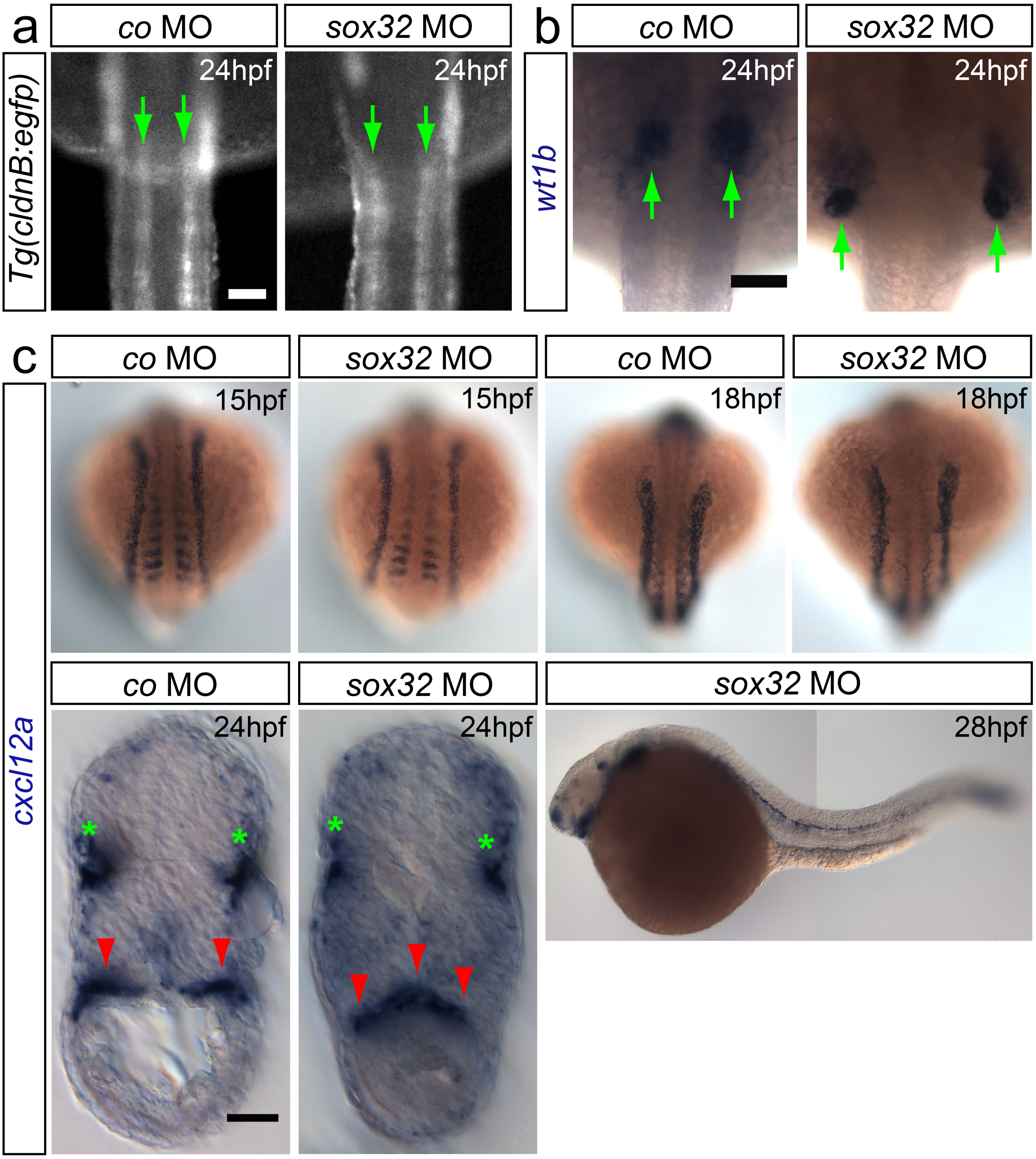

Supplementary Figure 13. Patterning of the mesodermal tissues adjacent to the gonad is normal in gut-deficient embryos

(**a**-**b**) Bilateral arrangement of pronephric ducts and the pronephros in embryos deficient for the gut is maintained (pronephric ducts labeled using (*Tg*(cldnB:egfp)) and pronephros with *wt1b* probe, green arrows). Anterior is up. (**c**) Bilateral *cxcl12a* expression is detected in the somites and lateral plate mesoderm of both control and gut-deficient embryos at 15 and 18hpf. At 24hpf *cxcl12a* expression at the gonad area is altered in embryos lacking the gut (additional red arrowhead in the cross-section). Green stars indicate *cxcl12a* expression in the lateral line. The expression of *cxcl12a* at the gonad region in embryos lacking the gut tube is no longer detected at 28hpf. Scale bars in all panels 50μm.

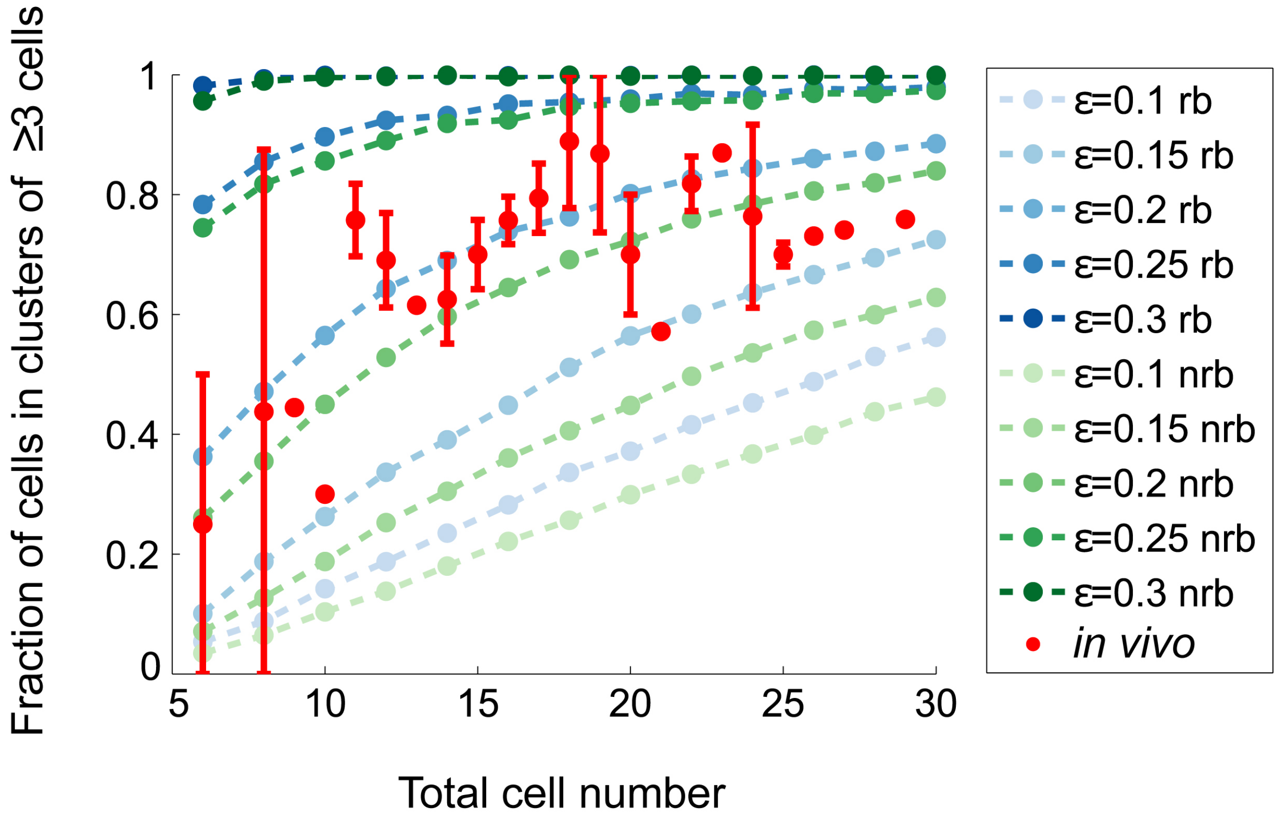

Supplementary Figure 14. Moderate levels of cell-cell adhesion result in cluster size distribution similar to *in vivo* values.

The fraction of cells in clusters composed of 3 or more cells as a function of the number of cells within the chamber (gonad region) is presented, for different strengths of cell-cell adhesion and boundary properties (reflective: rb (blue shades) and non-reflective: nrb (green shades)). The experimentally-measured values are presented in red (*in vivo*). The experimental measurements roughly match the simulation results when employing ε =0.2 regardless of the boundary conditions. Error bars for the *in vivo* values are the standard error of the mean, with very rare cases of cell number <11 and >26 having no error bars due to the fact that only one case per cell number was observed. Experimental data derived from 32 embryos (number of PGCs: n=1058).

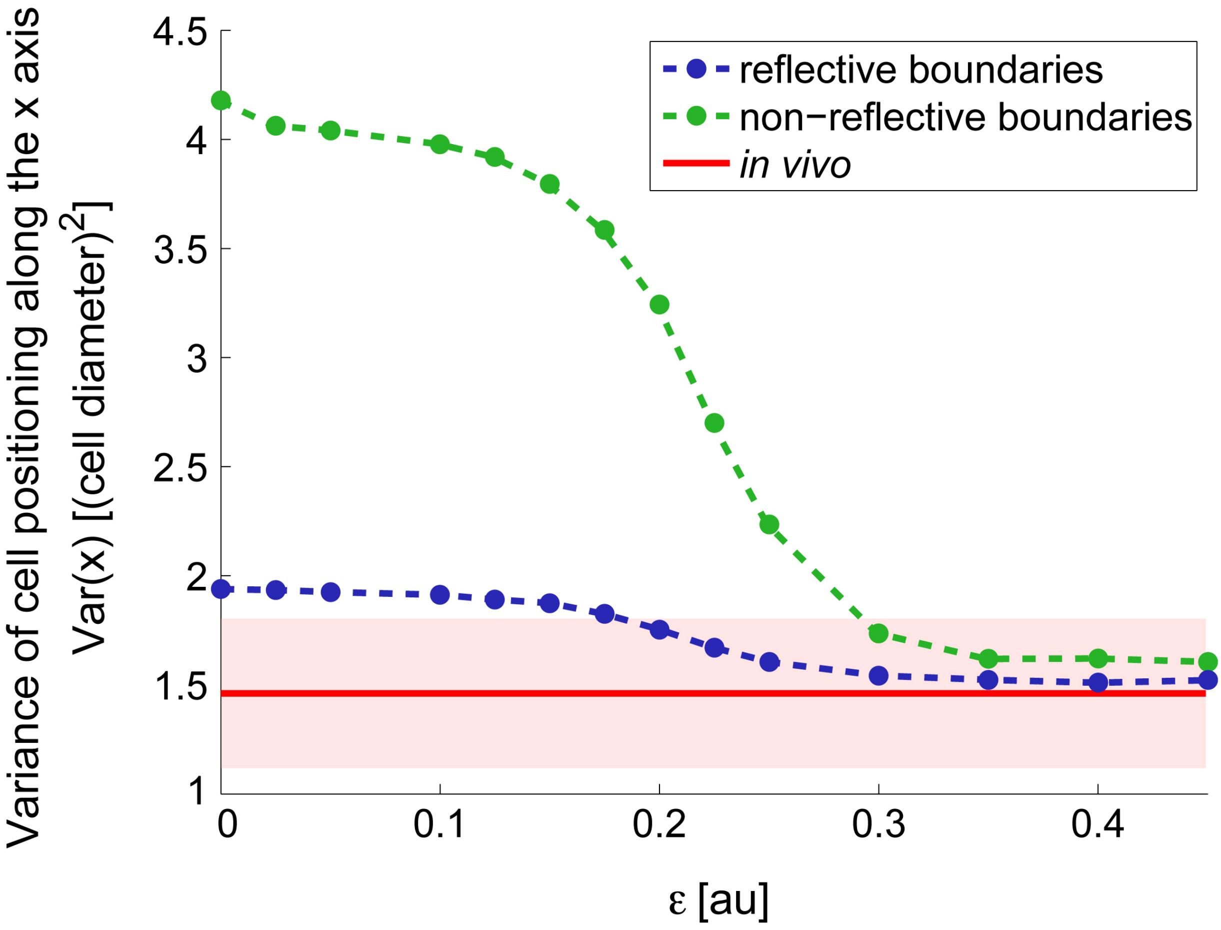

Supplementary Figure 15. Reflective boundary conditions contribute to central positioning of cell clusters.

Variance of the cell position along the width of the chamber (gonad region), as a function of cell-cell adhesion strength for non-reflective boundaries (green) and reflective boundaries (blue). The red line with pink error bar depicts the variance observed *in vivo* as measured in 5 embryos (number of PGCs: n=83). The error bar is the standard deviation of variances of cell position calculated for 10,000 samples bootstrapped from the data. When employing non-reflective boundaries, cells exhibiting low interaction level (small values of ε, Fig. 7a) accumulate at the boundaries, resulting in a large variance in their positioning across the chamber. When employing reflective boundaries, cells exhibiting low interaction level (small values of ε, Fig. 7b) are depleted from the boundaries, leading to a lower variance of in their positioning across the modeled area. The variance in the limit of non-adhesive particles (ε = 0) is estimated analytically in the Supplementary Note.

Supplementary Table 1. Experimental setup

Experiment	Fish lines used	Stage of treatment	Materials injected	Microscopy
20X image at 6hpf showing 4 PGC clusters (Fig. 1a)	*Tg(kop:egfp-f-3' nos3 UTR); Tg(ßactin:h2a-mcherry-3' globin UTR)*	-	-	Light-sheet
5X and 20X images at 24hpf showing lateral and dorsal views of PGC clusters (Fig. 1a)	*Tg(kop:egfp-f-3' nos3 UTR)*	1-cell	*mcherry-h2b*	Epifluorescence
5X image at 24hpf showing lateral view of the embryo (Fig. 1c)	*Tg(kop:egfp-f-3' nos3 UTR)*	1-cell	*mcherry-h2b*	SPIM (Single plane illumination microscopy)
10X movie showing a track of PGC at the gonad region (Fig. 1c; Supplementary Movie 2)	*Tg(kop:egfp-f-3' nos3 UTR)*	1-cell	*mcherry-h2b*	Two-photon
20X z-stacks of whole-mounts at 28hpf (*lpps* disruption)	*Tg(kop:egfp-f-3' nos3 UTR)*	1-cell	Control:<br>mixture of *co* sgRNAs	SPIM (Single plane illumination

(Fig. 3)			Experiment: mixture of *lpp* sgRNAs	microscopy)
20X z-stacks of whole-mounts at 28hpf (LPPs knockdown) (Supplementary Fig. 7)	*Tg(kop:egfp-f-3' nos3 UTR)*	1-cell	Control: 6 *co* MOs	SPIM (Single plane illumination microscopy)
			Experiment: 6 *lpp* MOs	
sgRNAs efficiency assay (Supplementary Fig. 4)	Wild-type	1-cell	Control: one *tyr* sgRNA	Light microscopy (Sequencing)
			Experiment: each *lpp* sgRNA	- (Sequencing)
5X images showing somatic phenotypes (*lpp*s disruption) (Supplementary Fig. 5a)	*Tg(kop:egfp-f-3' nos3 UTR)*	1-cell	Control: mixture of *co* sgRNAs	Epifluorescence
			Experiment: mixture of *lpp* sgRNAs	
5X images showing somatic phenotypes (LPPs knockdown) (Supplementary Fig. 5b)	*Tg(kop:egfp-f-3' nos3 UTR)*	1-cell	Control: 6 *co* MOs	Epifluorescence
			Experiment: 6 *lpp* MOs	
5X images showing MO efficiency (Supplementary Fig. 6a)	Wild-type	1-cell	each *MO-sensitive egfp* reporter and *mcherry-f-* / Control: *co* MO	Epifluorescence
			Experiment: each *lpp* MO	

<table>
<tr><td></td><td></td><td></td><td>3’globin UTR</td><td></td><td></td></tr>
<tr><td rowspan="3">10X images of whole embryos at 10hpf (LPPs gain-of-function) (Fig. 4a-c; Supplementary Fig. 8b)</td><td rowspan="3">med^{ny054}; Tg(kop:egfp-f-3’ nos3 UTR)</td><td rowspan="3">8-cell (into one of the blastomeres)</td><td colspan="2">Control:
pa-gfp-3’globin UTR</td><td rowspan="3">Epifluorescence</td></tr>
<tr><td colspan="2">Experiment:
lpp1-varX1-3’globin and lpp3-varX1-3’globin</td></tr>
<tr><td colspan="2">Rescue:
lpp3-varX1-H326K-H274K-3'globin UTR
and
lpp1-varX1-H326K-H274K-3'globin UTR</td></tr>
<tr><td rowspan="3">10X images of whole embryos at 10hpf (LPPs gain-of-function) (Supplementary Fig. 8a)</td><td rowspan="3">Tg(kop:egfp-f-3’ nos3 UTR)</td><td>1-cell</td><td colspan="2">cxcl12a MO
and
cxcl12b MO</td><td rowspan="3">Epifluorescence</td></tr>
<tr><td rowspan="2">8-cell (into one of the blastomeres)</td><td rowspan="2">mcherry-f-3’globin UTR</td><td>Control:
pa-gfp-3’globin UTR</td></tr>
<tr><td>Experiment:
lpp1-varX1-3’globin and lpp3-varX1-3’globin</td></tr>
<tr><td rowspan="3">Transplantation (LPPs gain-of-function) (Fig. 4d-e; Supplementary Fig. 9; Supplementary Movie 3)</td><td rowspan="2">Wild-type
(Donor)</td><td rowspan="2">1-cell</td><td rowspan="2">mcherry-f-3’globin UTR
and
cxcl12a-3’globin UTR</td><td>Control:
pa-gfp-3’globin UTR</td><td rowspan="3">Epifluorescence</td></tr>
<tr><td>Experiment:
lpp1-varX1-3’globin and lpp3-varX1-3’globin</td></tr>
<tr><td>med^{ny054};</td><td>4hpf: time of</td><td>-</td><td>-</td></tr>
</table>

	Tg(kop:egfp-f-3' nos3 UTR) **(Host)**	transplantation			
63X endodermal cell positioning (Supplementary Fig. 11e)	*Tg(kop:egfp-f-3' nos3 UTR)*	32 to 64-cell (into one of the blastomeres)	*taramA** and *secfp-3'globin UTR* and *mcherry-h2b*		Confocal
10X images of PGC granules (Supplementary Fig. 12e-f)	Wild-type	1-cell	*vasa-gfp-3'nos3 UTR*	Control: *co* MO	Epifluorescence
				Experiment: *sox32* MO	
40X z-stacks of 28hpf embryos showing the position of PGC clusters relative to the gut (Fig. 5b)	*Tg(sox17-dsred); Tg(kop:egfp-f-3' nos3 UTR)*	1-cell	Control: *co* MO		Confocal
			Experiment: *sox32* MO		
10X images of embryos rescued for the gut (Fig. 5c)	*Tg(kop:egfp-f-3' nos3 UTR)*	1-cell	*sox32* MO		Epifluorescence
		16-cell (into one of the corner blastomeres)	*mcherry-f-3'globin UTR*	Control: *pa-gfp-3'globin UTR*	
				Rescue: *sox17-3'globin UTR*	

20X images showing fusion of PGC clusters in $sox32^{ta56}$ embryos (Fig. 5d)	Control:<br>Wild-type<br>Experiment:<br>$sox32^{ta56}$	1-cell	*egfp-f-3'nos3 UTR*	Epifluorescence
10X dorsal movies of PGC clusters at the gonad region (Fig. 6a; Supplementary Movie 4)	*Tg(kop:egfp-f-3' nos3 UTR)*	1-cell	Control:<br>*co* MO<br>Experiment:<br>*sox32* MO	Epifluorescence
16X movies showing the behavior of PGC clusters relative to the gut (Fig. 6b; Supplementary Movies 1, 5 and 8)	*Tg(sox17-dsred); Tg(kop:egfp-f-3' nos3 UTR)*	-	-	SiMView
40X movies showing the polarity change in PGCs upon contacting the gut (Fig. 6c; Supplementary Movies 6, 7)	*Tg(sox17:egfp)*	1-cell	*lifeact-ruby-3'nos3 UTR*<br>and<br>*egfp-f-3'nos3 UTR*	SiMView

10X images of embryos showing correct mesodermal tissue development (Supplementary Fig. 13a)	*Tg(cldnB:egfp)*	1-cell	Control: *co* MO	Epifluorescence
			Experiment: *sox32* MO	

Supplementary Table 2. Expression of *s1pr* and *lpar* RNAs in PGCs
A list of RNAs encoding for S1P and LPA receptors and their expression levels (mean counts obtained in RNA-seq analysis) in PGCs isolated from 7hpf and 36hpf embryos.

**Transcript**	**RNA-seq on PGCs**	
	**7hpf**	**36hpf**
s1pr2	190.3	641.9
s1pr5a	44.1	384.9
lpar2a	97.9	239.3
lpar2b	208.2	220.2
lpar1	42.9	150.8
s1pr1	2.8	31.1

Supplementary Table 3. Single-guide RNAs used

sgRNA (Internal number)	Sequence 5'-3' (gene-specific region is underlined)	Amount injected per embryo (pg)	Reference
tyr-sgRNA1-exon1 (G416)	ATTTAGGTGACACTATAGAACTGGAGGACTTCTGGGGGTTTTAGAGCTAGAAATAGCAAG	700pg for mutation detection; 55	[12]
tyr-sgRNA2-exon1 (G417)	ATTTAGGTGACACTATAGACTGATCAGGCTGCGGTTGGTTTTAGAGCTAGAAATAGCAAG	55	-
tyr-sgRNA3-exon2 (G418)	ATTTAGGTGACACTATAGACCGTCACACAAAGCCTCGGTTTTAGAGCTAGAAATAGCAAG	55	-
tyr-sgRNA4-exon4 (G419)	ATTTAGGTGACACTATAGAGTCTCCGTTCCTGTAGAGGTTTTAGAGCTAGAAATAGCAAG	55	-
alb-sgRNA1-exon1 (G420)	ATTTAGGTGACACTATAGAGGAGTGGTGGAGCCTCCGGTTTTAGAGCTAGAAATAGCAAG	55	-
alb-sgRNA2-exon1 (G421)	ATTTAGGTGACACTATAGAAATTCTGCTACGCTGTTGGTTTTAGAGCTAGAAATAGCAAG	55	-
alb-sgRNA3-exon2 (G422)	ATTTAGGTGACACTATAGAGTCCGTCAATGAAGTCTGGTTTTAGAGCTAGAAATAGCAAG	55	-
alb-sgRNA4-exon4 (G423)	ATTTAGGTGACACTATAGATGAGTCATGCAGAGACAGGTTTTAGAGCTAGAAATAGCAAG	55	-
gol-sgRNA1-exon1 (G424)	ATTTAGGTGACACTATAGAACAGACGTGTTTCTCCAGGTTTTAGAGCTAGAAATAGCAAG	55	-
gol-sgRNA2-exon2 (G425)	ATTTAGGTGACACTATAGATTCTTCACGGTGCAGGAGGTTTTAGAGCTAGAAATAGCAAG	55	-
gol-sgRNA3-exon3 (G426)	ATTTAGGTGACACTATAGATCTCTCGCAGGATGTTGCGTTTTAGAGCTAGAAATAGCAAG	55	[12]
gol-sgRNA4-exon4 (G427)	ATTTAGGTGACACTATAGAGTGCATCTGTGCAGCGTGGTTTTAGAGCTAGAAATAGCAAG	55	-
lpp1-sgRNA1-exon1 (G045)	ATTTAGGTGACACTATAGATAAGATGTTTGAAACCAGGTTTTAGAGCTAGAAATAGCAAG	25	-
lpp1-varX1-sgRNA2-	ATTTAGGTGACACTATAGACTGCTGTGGAAAGGGTAGGTTTTAGAGCTAGAAATAG	25	-

sgRNA (Internal number)	Sequence 5’-3’ (gene-specific region is underlined)	Amount injected per embryo (pg)	Reference
exon2 (G046)	CAAG		
lpp1-varX2-sgRNA2-exon2 (G047)	ATTTAGGTGACACTATAGATCAAGTACCCATATAAAGGTTTTAGAGCTAGAAATAGCAAG	700 for mutation detection; 25	-
lpp1-sgRNA3-exon3 (G048)	ATTTAGGTGACACTATAGAGCACACATCCAGGAAGTGGTTTTAGAGCTAGAAATAGCAAG	25	-
lpp1-sgRNA4-exon5 (G049)	ATTTAGGTGACACTATAGATCATCCAAGGAGCCATCGGTTTTAGAGCTAGAAATAGCAAG	25	-
lpp1-like-sgRNA1-exon1 (G050)	ATTTAGGTGACACTATAGACAGAATGTTCGAGGCTCGGTTTTAGAGCTAGAAATAGCAAG	25	-
lpp1-like-sgRNA2-exon2 (G051)	ATTTAGGTGACACTATAGAGCACAGACCTTTCAAAAGGTTTTAGAGCTAGAAATAGCAAG	25	-
lpp1-like-sgRNA3-exon3 (G052)	ATTTAGGTGACACTATAGACCTGCTTTACAGTCGATGGTTTTAGAGCTAGAAATAGCAAG	25	-
lpp1-like-sgRNA4-exon5 (G053)	ATTTAGGTGACACTATAGATATCTGCAGTCCAGAATGGTTTTAGAGCTAGAAATAGCAAG	25	-
lpp2-sgRNA1-exon1 (G062)	ATTTAGGTGACACTATAGAAGAAACTCTTCGTCCTGGGTTTTAGAGCTAGAAATAGCAAG	700 for mutation detection; 25	-
lpp2-sgRNA2-exon2 (G260)	ATTTAGGTGACACTATAGAAATAGTCATTGCCACAAAGTTTTAGAGCTAGAAATAGCAAG	25	-
lpp2-sgRNA3-exon3 (G261)	ATTTAGGTGACACTATAGACAAACTGCTAGGAAATTGGTTTTAGAGCTAGAAATAGCAAG	25	-
lpp2-sgRNA4-exon5 (G262)	ATTTAGGTGACACTATAGAAAATGGGCTCGTCTCCTGGTTTTAGAGCTAGAAATAGCAAG	25	-
lpp2-like-sgRNA1-exon1 (G263)	ATTTAGGTGACACTATAGAATCTTCTGTGTGTCGCCGGTTTTAGAGCTAGAAATAGCAAG	25	-
lpp2-like-sgRNA2-exon2 (G264)	ATTTAGGTGACACTATAGAATGGTGTCTGGTCTGTAGGTTTTAGAGCTAGAAATAGCAAG	700 for mutation detection; 25	-
lpp2-like-sgRNA3-	ATTTAGGTGACACTATAGATGTCGGTTTGTGCTCCAGGTTTTAGAGCTAGAAATAGC	25	-

sgRNA (Internal number)	Sequence 5'-3' (gene-specific region is underlined)	Amount injected per embryo (pg)	Reference
exon3 (G265)	AAG		
lpp2-like-sgRNA4-exon5 (G266)	ATTTAGGTGACACTATAGAAACACCACTGGAGTGACGGTTTTAGAGCTAGAAATAGCAAG	25	-
lpp3-sgRNA1-exon1 (G054)	ATTTAGGTGACACTATAGAACCCGAAACTAGAAACGGGTTTTAGAGCTAGAAATAGCAAG	25	-
lpp3-sgRNA2-exon2 (G055)	ATTTAGGTGACACTATAGATGACAATTAGAATGCCAGGTTTTAGAGCTAGAAATAGCAAG	25	-
lpp3-sgRNA3-exon3 (G056)	ATTTAGGTGACACTATAGAACAAACGTCTAGGAAGTGGTTTTAGAGCTAGAAATAGCAAG	25	-
lpp3-sgRNA4-exon5 (G057)	ATTTAGGTGACACTATAGAGTCTCGATTCACATGGCGGTTTTAGAGCTAGAAATAGCAAG	25	-
ppap2d-varX2-sgRNA1-exon1 (G058)	ATTTAGGTGACACTATAGATGACAGCCTGGATAGATGGTTTTAGAGCTAGAAATAGCAAG	700 for mutation detection; 25	-
ppap2d-varX1-sgRNA1-exon1 (G059)	ATTTAGGTGACACTATAGAAATTCGGAGCTCCAGCAGGTTTTAGAGCTAGAAATAGCAAG	25	-
ppap2d-sgRNA2-exon2 (G060)	ATTTAGGTGACACTATAGAGGTGACACCATACATGAGGTTTTAGAGCTAGAAATAGCAAG	25	-
ppap2d-sgRNA3-exon3 (G061)	ATTTAGGTGACACTATAGATAGAGACAGGACACGTAGGTTTTAGAGCTAGAAATAGCAAG	25	-

Supplementary Table 4. Morpholino Antisense Oligonucleotides used

Morpholino (Internal number)	Sequence 5'-3'	Concentration (µM) (1nl injected)
co MO (1)	CCTCTTACCTCAGTTACAATTTATA	Identical to the experimental MO
cxcr4b-mismatch MO (9); used as a *control* MO in the mixture	AATTGAAGCTATCGTAATATTGCAT	75
lpp3-mismatch MO (206); used as a *control* MO in the mixture	ACAGTTGCTTCTCATGTGTTTTTGC	75
lpp1-mismatch MO (207); used as a *control* MO in the mixture	ATGATCTCTCATGCAAAAGCAACCA	75
Xenopus laevis p53 MO (208); used as a *control* MO in the mixture	GCCGGTCTCAGAGGAAGGTTCCATT	75
gal4-uas MO (209); used as a *control* MO in the mixture	GTTCGATAGAAGACAGTAGCTTCAT	75
lpp1 MO (144)	ATCATCTGTGATGGAAAAGGAAGCA	75
lpp1-like MO (130)	GCTTCTGCTCATCAAACTGCTGAAC	75
lpp2 MO (131)	GTTTCTTTTTCCCCATGTCCGACAT	75
lpp2-like MO (132)	TTCTGTTCTTCTTCAGCTCCGACAT	75
lpp3 MO (99)	AGAGTTCCTTCTGATGTCTTTTTCC	75
ppap2d MO (116)	AATTCAACTTCTGCATGATGTCCGT	75
cxcl12a MO (12)	TTGAGATCCATGTTTGCAGTGTGAA	200
cxcl12b MO (13)	GCGCTACTACTTTGCTATCCATGCC	200
sox32 MO (71)	CAGGGAGCATCCGGTCGAGATACAT	200

Supplementary Table 5. *sox32* expression in PGCs and somatic cells
sox32 expression level (mean counts obtained in RNA-seq analysis) in PGCs and all somatic cells of the embryo at 7 and 36hpf.

	**RNA-seq**			
	**7hpf**		**36hpf**	
sox32	PGCs	Soma (total embryo)	PGCs	Soma (total embryo)
	268.5	422.6	2132.9	3213.3

Supplementary Table 6. Expression of cell adhesion molecules in PGCs
A list of RNAs encoding for cell adhesion molecules that exhibit enhanced expression levels (mean counts obtained in RNA-seq analysis) in PGCs at 36hpf compared to 7hpf. Transcripts with expression levels higher than 14 mean counts at 36hpf are listed.

**Transcript**	**RNA-seq**	
	PGCs	
	7hpf	36hpf
cadherin 2 (n-cadherin)	3879.4	12697.7
cadherin 6	125.5	3582.7
protocadherin 19	262.6	2335.1
protocadherin 8	508.3	1286.8
cadherin 1 (e-cadherin)	278.7	1249.7
protocadherin 18b	206.3	1022.3
protocadherin 10b	133.1	976.4
cadherin 11	13.5	105.2
cadherin 23	21.7	77.5
protocadherin 2 gamma 8	2.4	74.3
cadherin 17	9.5	38.6
protocadherin 1 gamma 22	0.0	36.6
protocadherin 1 gc 6	2.0	34.3
protocadherin 1 gamma 26	3.9	23.9
protocadherin 15a	4.8	22.1
cadherin 7	2.7	16.9
protocadherin 1 gamma 32	0.9	14.1

Supplementary Table 7. Fish lines used

Fish line name	Reference
wild-type of the AB background	-
Tg(kop:egfp-f-3'nos3 UTR)	[2]
Tg(kop:mcherry-f-3'nos3 UTR)	[3]
Tg(βactin:h2a-mcherry-3'globin UTR)	[4]
Tg(sox17:egfp)^{s870}	[5]
Tg(sox17:dsred)^{S903}	[5]
Tg(cldnB:egfp)	[6]
med^{ny054}	[7]
clo^{m39}	[8]
flh^{tk241}	[9]
sox32^{ta56}	[10]
ody^{t26035}	[11]

Supplementary Table 8. Constructs cloned, primers used for cloning , RNA amount injected per embryo and MO efficiency PCR-based test

Construct (internal number)	F-primer, 5'-3' (internal number)	R-primer, 5'-3' (internal number)	Accession number	RNA amount injected (pg)
lpp1-varX1-3'globin UTR (C043)	AAAGGATCCACC ATGTTTGAAACCA GAGGAATCCCTTT TATATTG (D928)	AAACTCGAGTTAGT TCGGGTTATCGTA ATGATTTCCATTTG (D929)	XM_692415.7	50
lpp3-varX1-3'globin UTR (B682)	AAAAAGCTTACCA TGCAAAACTATAT GTATGAGAAGGC G (D112)	AAAAAGCTTCTACA CCATGTTGTGATG GTTGTTC (D113)	XM_001919526. 2 template: B690	50
lpp3-varX1-H326K-3'globin UTR (C460)	CTGATCACAAGAA GCACCCCACTGA TG (E469)	CATCAGTGGGGTG CTTCTTGTGATCAG (E470)	XM_001919526 Template: B682	intermediate construct for C488
lpp3-varX1-H326K-H274K-3'globin UTR (C488)	CCTTCTTTTCCGG AAAGGCTTCATTT TC (E467)	GAAAATGAAGCCT TTCCGGAAAAGAA GG (E468)	XM_001919526 template: C460	50
lpp1-varX1-H326K-3'globin UTR (C456)	TGTCAGACTACAA AAAGCACTGGAG TGACGT (E465)	ACGTCACTCCAGT GCTTTTTGTAGTCT GACA (E466)	XM_692415.7 template: C043	intermediate construct for C486
lpp1-varX1-H326K-H274K-3'globin UTR (C486)	CATTCTATTCAGG CAAGTCCTCGTTC TCC (E463)	GGAGAACGAGGAC TTGCCTGAATAGAA TG (E464)	XM_692415.7 template: C456	50
sox17-3'globin UTR (B624)	AAACCATGGACC ATGAGCAGTCCC GATGCGG (D130)	AAAACTAGTTCAAG AATTATTATAGCCG CAGTAGTATACAG C (D131)	NM_131287	100
lpp1-varX1 for *in situ* probe (C404)	TGATCTCCCGCTT GCAGCGTTTA	CTACATATGGGAA GTGTGAAGCCCAC	XM_692415.7	-

	(D930)	TGT (E409)		
lpp1-varX2 for *in situ* probe (C405)	GGACTCCCATTTGCAATACTCACAATACAAC (D931)	GTAAGCAAAGTGACAGGGATCATAACTCCTC (E410)	XM_005155458.2	-
lpp1-like for *in situ* probe (B692)	AAAAAGCTTACCATGTTCGAGGCTCGAGGACTCT (D120)	AAAAAGCTTTCACTGTGTGCTGCCATAGTGG (D121)	XM_001334553.4	-
lpp2 for *in situ* probe (B693)	AAAAAGCTTACCATGGGGAAAAAGAAACTCTTCGTC (D122)	AAAAAGCTTTCACACAGGTCCAGAATAGCGG (D123)	NM_199953.1	-
lpp2-like for *in situ* probe (B694)	AAAGGATCCACCATGCCATTCATCATAATGACCATCA (D162)	AAACTCGAGTCAAGCGGTGGTTTTAAGTTTGTC (D125)	XM_687169.7	-
lpp3-varX1 for *in situ* probe (B690)	AAAAAGCTTACCATGCAAAACTATATGTATGAGAAGGCG (D112)	AAAAAGCTTCTACACCATGTTGTGATGGTTGTTC (D113)	XM_001919526.2	-
ppap2d for *in situ* probe (B691)	AAACCATGGACCATGCAGAAGTTGAATTCGGTGAAC (D118)	AAAACTAGTTTAGCAGACGGTCTGCTGGCT (D119)	NM_001079978.1	-
MO binding site-lpp3-varX1-egfp-globin 3'UTR (B833)	AAACTCGAGGACCACTGTGCAAAACAAATATCTGGG (D354)	AAAGGTACCCCACCATGTTGTGATGGTTGTTCCTCTC (D355)	XM_001919526.2	100
MO binding site-ppap2d-egfp-globin 3'UTR (B938)	AAACTCGAGATTGAATTTTACGGACATCATGCAGAAGTTGAATTCGGTG	AAAGGTACCCGCAGACGGTCTGCTGGC (D590)	NM_001079978.1	100

	(D589)			
MO binding site-lpp1-like-egfp-globin 3'UTR (B949)	AAAGGATCCACCATGTTCAGCAGTTTGATGAGCAGAAGC (D620)	AAAGGATCCCCCTGTGTGCTGCCATAGTGGTTTGG (D621)	XM_001334553.4	100
MO binding site-lpp2-egfp-globin 3'UTR (D596)	CTTCCAAGTGGATTGCGTTT (G438); Nested PCR using: GATCACTCGAGACCATGGGGAAAAAGAAACTC (G436)	TGCGAGTATTCAACCTCATTTC (G439); Nested PCR using: GATCTGGTACCCCACAGGTCCAGAATAGC (G437)	NM_199953.1	100
MO binding site-lpp2-like-egfp-globin 3'UTR (C005)	AAAGGATCCACCATGTCGGAGCTGAAGAAGAACAGAATG (D876)	AAAGGATCCCCTACAGGCCTGCTGATTGGATAATAGTTG (D877)	XM_687169.7	100
lpp1 amplification for *lpp1* splice-blocking MO efficiency test	ATGTTTGAAACCAGAGGAATCCCTTTTATATTG (D926)	TTAGTTCGGGTTATCGTAATGATTTCCATTTG (D927)	-	-
odc amplification as a negative control for *lpp1* splice-blocking MO efficiency test	GTTTGCAGGCTGAGTGTGAA (A451)	CGCATGATCATACAGGATGC (A452)	-	-

Supplementary Table 9. Additional constructs used

Construct	RNA amount injected (pg) per embryo
mcherry-h2b (B325)	100;<br>150 for YSL nuclei labelling
mcherry-f-3'globin UTR (A709)	70;<br>30 for MO efficiency
pa-gfp-3'globin UTR (A918)	Identical to the experimental RNA
cxcl12a-3'globin UTR (642)	15
*taramA** (activated) (66)	5
secfp-3'globin UTR (B758)	40
vasa-gfp-3'nos3 UTR (534)	100
egfp-f-3'nos3 UTR (493)	60
lifeact-ruby-3'nos3 UTR (B007)	120
cxcl12a for *in situ* probe (607)	-
cxcl12b for *in situ* probe (609)	-
nos3 for *in situ* probe (651)	-
foxa3 for *in situ* probe (45)	-
kdrl for *in situ* probe	-
sox17 for *in situ* probe (206)	-
foxa1 for *in situ* probe (205)	-
piwil1 for *in situ* probe (A505)	-
wt1b for *in situ* probe (75)	-

Supplementary Table 10. RNAscope probes used

**Probe Name (Internal Number)**	**Accession Number**	**Selected region**	**Catalog number**
dapB (1)	EF191515	414-862 bp	310043
myoD (5)	NM_131262.2	2-1083 bp	402461-C2
cxcl12a (16)	NM_178307.2	24-1508 bp	406481-C2
nos3 (18)	NM_131878.1	30-509 bp	431191-C3
egfp (13)	U55763.1	16-740 bp	400281

Supplementary Note

Calculation of the position distribution for non-interacting particles

Using the simulations in the two-dimensional chamber, under conditions of no adhesion ($\epsilon = 0$), we have obtained substantial differences in the distribution of the particles for the two boundary conditions (reflective and non-reflective) (Supplementary Fig. 15). Since the two-dimensional model is not amenable to analytic description, to gain a deeper quantitative understanding we investigated the behavior of a one-dimensional representation (projection) of the full two-dimensional system. This is described below in this supplementary note.

1 Mapping the 2D system to a 1D approximate model

We attempt to approximate the distribution of the particles along the width of the 2D channel, using a 1D analytical model, for the case of non-interacting particles ($\epsilon = 0$). We show that the variance of the obtained approximate distributions for both reflective and non-reflective boundaries is comparable to those of the 2D simulations (Supplementary Fig. 15).

The two dimensional simulation of the particles, is as follows: Each particle moves due to a constant magnitude motility force, whose direction diffuses with time. The equation of motion of each particle is the overdamped Langevin equation:

$$\partial_t \vec{r} = v_0 \hat{n}_\theta(t) \tag{1a}$$

$$\partial_t \theta = \eta(t) \tag{1b}$$

where v_0 is the self propulsion velocity magnitude, $\vec{r} = (x, y)$ is the particle's position, $\hat{n}_\theta = (\cos\theta, \sin\theta)$ is a unit vector in the direction of the motility force of the particle, and η is white noise obeying the relations $\langle \eta(t) \rangle = 0$, $\langle \eta(t)\eta(t') \rangle = 2D_r \delta(t - t')$.

The long axis of the channel geometry used in the simulation is periodic and denoted as the y direction, while in the x direction the region is bounded by hard walls. Since the system is symmetric to translation in the y direction, the particle density is independent of y. We are interested in finding the steady state density of particles along the width of the channel (x). Projecting equation (1a) onto the x axis yields:

$$\partial_t x = v_0 \cos\theta(t) = v_0 |\cos\theta(t)| \text{sign}(\cos\theta(t)) \tag{2}$$

Next, we perform an approximation, and replace $|\cos\theta|$ by its average over all angles $\langle |\cos\theta| \rangle = \frac{2}{\pi}$ (similar to [13]), to write an approximate one dimensional Langevin equation

$$\partial_t x = v_1 m(t) \tag{3}$$

where $v_1 = 2v_0/\pi$ and $m(t)$ stochastically changes value from 1 to -1 and vice versa according to the direction of the particle's motion along the x-axis.

The statistics of the flipping times of $m(t)$ (in which $m(t)$ changes sign) are given by the first-passage problem for the angle θ in the interval $[0, L]$, where $L = \pi$, with absorbing boundaries. The solution for this problem is found in [14]. The survival probability is dominated by the slowest decaying eigenmode: $S(t) \propto e^{-\frac{t}{\tau_1}}$, with $\tau_1 = \frac{L^2}{D_r \pi^2} = 1/D_r$. Thus the probability that absorption at one of the boundaries had already occurred at time t ($m(t)$ changed sign) is $1 - S(t)$. Hence the flipping times of $m(t)$ are approximately exponentially distributed with a mean flipping time $1/D_r$.

2 Non-reflective boundaries

In this case, particles that hit the boundary remain at the boundary until their motility force rotates away. This causes a discontinuity in the particle density at the boundaries, where particles accumulate. We calculate the density profile of the particles using the one dimensional approximate model. The rate equations which describe the process of particle motion and direction changes of Eq. 3, are the following[15, 16]: For $-d < x < d$,

$$\begin{aligned} \partial_t R &= -v_1 \partial_x R + D_r(L - R) \\ \partial_t L &= v_1 \partial_x L + D_r(R - L) \end{aligned} \tag{4}$$

where $R(x,t), L(x,t)$ are the local densities of the right/left moving particles respectively. At the boundaries (at $x = \pm d$), we have the following equations for the number of accumulated particles:

$$\begin{aligned} & x = -d: \\ & \partial_t N_L^{-d} = -J_L(-d) - D_r N_L^{-d} \\ & \partial_t N_R^{-d} = -J_R(-d) + D_r N_L^{-d} \\ & x = d: \\ & \partial_t N_L^{d} = J_L(d) + D_r N_R^{d} \\ & \partial_t N_R^{d} = J_R(d) - D_r N_R^{d} \end{aligned} \tag{5}$$

where N_R^d is the number of particles at $x = d$ with self-propulstion velocity pointing right, and the notations N_L^{-d}, N_L^d, N_R^{-d} are defined similarly. We also define $J_R(x) = v_1 R(x)$ as the current of right moving particles, and $J_L(x) = -v_1 L(x)$ as the current of left moving particles.

The number of particles at the boundaries which move towards the boundary: N_L^{-d}, N_R^d increases due to the incoming flux from the bulk, and decreases due to motor flipping of the particles. Since particles whose direction of motion is away from the boundary do not accumulate at the boundary, $N_L^d = N_R^{-d} = 0$. The only extra equation needed to solve for the steady-state particle densities is the conservation of total particle number: $\int_{-d}^{d} \rho(x)\, \mathrm{d}x + N^{-d} + N^d = 1$ (where $N^{\pm d} = N_L^{\pm d} + N_R^{\pm d}$, and $\rho(x) = R(x) + L(x)$ is the total particle density in the bulk).

The steady-state solution is:

$$L(x) = R(x) = \frac{1}{2}\frac{D_r}{2dD_r+v_1}$$

$$N_L^{-d} = N_R^{d} = \frac{v_1}{2(2dD_r+v_1)} \tag{6}$$

We compare this approximate solution to the simulated particle density distribution from the 2D model in Fig. I. The variance of the distribution for the 2D simulation is 4.18, while for the 1D approximate model we get a variance of 3.98. When comparing to the variance difference between the two boundary conditions, shown in Supplementary Fig. 15, we conclude that the 5% difference between the 2D and 1D models is indeed very small, demonstrating that the 1D approximation provides a reasonable quantitative description.

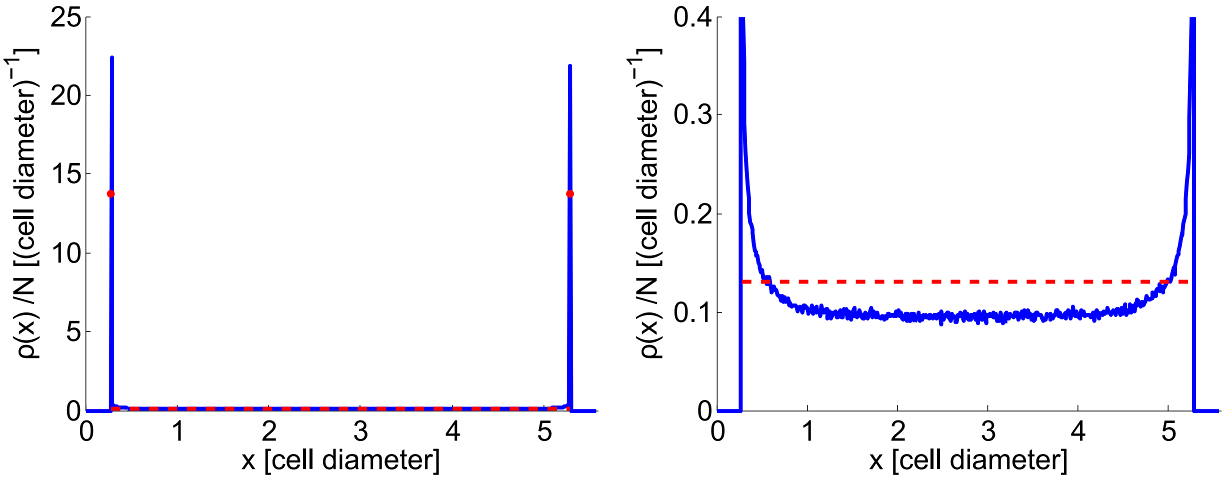

(a) particle density

(b) particle density - zoom in

Figure I: The particle density distribution along the x axis for the case of non-reflective boundaries, $\epsilon = 0$: blue - simulation result, dashed red - 1D approximation. The model parameters used: $D_r = 1/60$ min^{-1}, $v_0 = 0.0545$ cell diameter/min, $v_1 = \frac{2}{\pi}v_0$ cell diameter/min, $d = 2.78$ cell diameters. The red dots in (a) denote the binned accumulation at the boundaries calculated from the 1D model.

3 Reflective boundaries

Here we treat the system with reflecting boundaries using our 1D approximate model. When particles hit the boundary in the 2D simulation, they reorient and leave the boundary in the perpendicular direction. Thus, while particles have an average velocity of $v_1 = 2v_0/\pi$ along the x-axis, they have a faster x-velocity of v_0 after being reflected. Such fast (reflected) particles slow down to the normal average x-velocity due to reorientation of their direction of motion: the initial θ at the boundary is perpendicular, and it needs to change by $\pi/4$ to either side for the particle recover the average x-velocity. This gives an interval of size $\pi/2$ in which again we consider a first-passage problem with absorbing boundaries, such that now: $\tau_1 = \frac{1}{4D_r}$. In

this system we expect the density of particles to be close to uniform, and depleted near the boundary over a lengthscale $\sim v_0 D_r$.

Similar to the case of non-reflective boundaries, we write the 1D equations (as in Eqs. 4), where we add new types of particles that move fast, with velocity v_0, while the velocity of the "regular" particles is v_1. A left moving particle (fast or regular) becomes a fast right moving particle upon hitting the left boundary, and similarly for right moving particles hitting the right boundary. Fast particles become regular ones which move in the same direction at rate $aD_r = 1/\tau_1$, due to the reasoning above.

The equations are therefore:

$$\begin{aligned} \partial_t R &= -v_1 \partial_x R + D_r(L - R) + aD_r R_f \\ \partial_t L &= v_1 \partial_x L + D_r(R - L) + aD_r L_f \\ \partial_t R_f &= -v_0 \partial_x R_f - aD_r R_f \\ \partial_t L_f &= v_0 \partial_x L_f - aD_r L_f \end{aligned} \tag{7}$$

where $R(x,t), L(x,t)$ are the local densities of the regular right/left moving particles respectively, and $R_f(x,t), L_f(x,t)$ are the local densities of the faster right/left moving particles that emerge from the reflecting boundaries, respectively. We seek a steady state solution for the particle densities. Equating all RHSs of the equations in (7) to zero, a system of four linear first order differential equations is obtained. Its general solution contains four constants to be determined by the boundary conditions. On the left boundary ($x = -d$), left moving particles become fast right moving particles. Thus the flux of left moving particles is equal to the flux of fast right moving particles at $-d$. A second boundary condition on the left boundary is that right moving particles do not cross the boundary, thus their flux there is zero. These two conditions along with the mirror-symmetrical ones for the right boundary $x = d$ give us the following four boundary conditions:

$$\begin{aligned} &v_1 L(-d) + v_0 L_f(-d) = v_0 R_f(-d) \\ &R(-d) = 0 \\ &v_1 R(d) + v_0 R_f(d) = v_0 L_f(d) \\ &L(d) = 0 \end{aligned} \tag{8}$$

Due to the symmetry of the problem, one of the above conditions is redundant, as it is automatically satisfied when the other three are imposed. Thus we have a remaining constant in the solution, which is determined by the normalization condition:

$$\int_{-d}^{d} dx \rho(x) = 1 \tag{9}$$

where we define the total particle density $\rho(x) \equiv L(x) + R(x) + L_f(x) + R_f(x)$. The solution of Eq. (7) with boundary conditions (8) and total particle number conservation normalization condition (9) is:

$$\rho(x) = \frac{aD_r}{v_0} \left(A \cosh \left(\frac{aD_r}{v_0} x \right) + B \right) \tag{10}$$

where

$$
\begin{aligned}
A &= (av_1^2 - av_1v_0 - 2v_0^2)e^{\frac{aD_r}{v_0}d}/C \\
B &= \left((av_1v_0 + v_0^2)e^{\frac{2aD_r}{v_0}d} + v_0^2\right)/C \\
C &= -av_1^2 + av_1v_0 + 2v_0^2 + 2adD_rv_0 + e^{\frac{2aD_r}{v_0}d}\left(2adD_r(av_1 + v_0) + av_1^2 - av_1v_0 - 2v_0^2\right)
\end{aligned}
\tag{11}
$$

The density is maximal in the center, and decreases to minima at the edges due to the depletion caused by the reflective boundaries (Fig. II). This reproduces the effect that appears in the simulated 2D system, where a depletion zone with decreased density appears near the edges.

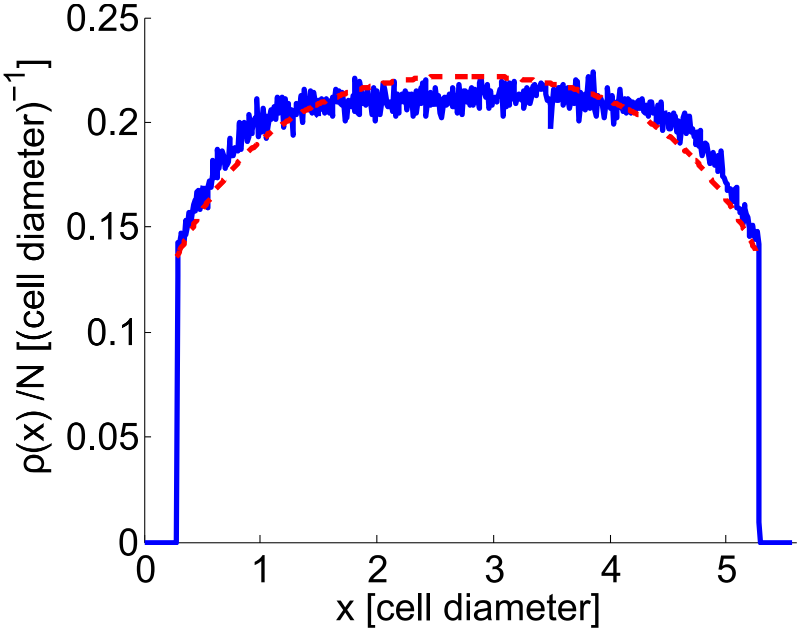

Figure II: The particle density distribution along the x axis for $\epsilon = 0$, in the case of reflecting boundaries: blue - simulation result, dashed red - 1d approximation. The model parameters used: $a = 4$, $D_r = 1/60$ min^{-1}, $v_0 = 0.0545$ cell diameter/min, $v_1 = \frac{2}{\pi}v_0$ cell diameter/min, $d = 2.5$ cell diameters.

We next calculate the variance of the density distribution from the 1D approximate model and compare it to the 2D simulation result. The variance obtained from the 2D simulation is 1.94, while in the 1D model the variance is 1.87. For comparison we note that the variance of a uniform distribution in the same interval is 2.08. We therefore see that the main effect of the reflective boundary on the variance of the density distribution is due to the removal of the particle accumulation at the boundary.

Supplementary Method 1

Two color in situ protocol, See Reference 30 for more details.

General:

Principle of double staining:

A mix of different Dig- labeled and Fluo-labeled probes is used for hybridization.

The antibody-and subsequent staining steps are performed separately for both types of probes. After completion of the first staining reaction the first antibody is removed with acidic solution, followed by the second antibody incubation. The reason for doing so is that both antibodies are coupled to the same enzyme (alkaline phosphatase). This enzyme can be used with different dye combinations, here X-phosphate/NBT and X-phosphate/ INT.

Problems/ considerations:

1 The second staining step is generally less sensitive and, for some probes (like vasa) less reliable than the first one.

2 Fluo-probes tend to be more problematic than Dig-probes. (**Fluo-probes often give a weaker signal and more background**)

3 Doing the blue staining as a first step has the advantage that the embryos can be cleared in EtOH before the second step. This can strongly reduce dark background in the yolk.

As both antibodies are coupled to the same enzyme cross reactions during the staining take place. Therefore you have to do the blue staining first to avoid unspecific blue staining on top of the red staining.

All principle combinations of probe, dye and first or second step are possible. The best combination should be chosen according to the above mentioned considerations.

For second step staining the probe concentration should be relatively high. For Fluo probes the concentration should be adjusted carefully.

For blue staining as a first step the probe concentration should be low in order to get minimum background.

Examples for probe concentrations: see below

In the following protocol, the first step is blue staining for DIG probes.

Fixation and storage of embryos:

- **embryos younger than 24 hrs**: fix overnight (not longer than 24 h) at 4°C in 4% PFA in PBS, then rinse 3 times with PBTand then dechorionate by hand/ pronase before transferring to MetOH.
- **embryos 24hrs and older**: remove chorion before fixation by hand/ pronase.
  Fix overnight at 4°C in 4% PFA in PBS and then rinse 3 times with PBT before transferring to MetOH.

- **Embryos younger than 1-2 S, have to be fixed for 2 days!**

Transfer to MetOH:

- 25% MetOH - 75% PBT for 5 min
- 50% MetOH - 50% PBT for 5 min
- 75% MetOH - 25% PBT for 5 min
- 100% MetOH, replace with fresh MetOH 100% after 5 min

Embryos can be stored in 100% MetOH at –20°C for several months.

In situ Day 1:

- **Rehydration**:

Transfer embryos into 1.5 ml tubes and rehydrate by successive incubations in:

- 75% MetOH - 25% PBT for 5 min
- 50% MetOH - 50% PBT for 5 min
- 25% MetOH - 75% PBT for 5 min
- 100% PBT 4 x 5 min

(add solutions with a 10ml pipette. Pre-gastrula-embryos are fragile during rehydration - add solutions slowly. Make sure that pH of 1x PBT is 7.4)

- **Proteinase K:**

Digest with Proteinase K (5 µg/ml in PBT, 1:2000 dilution):

During the incubation, we lay the tubes on their sides to let the embryos be exposed to Proteinase K equally.

- for very early stages the Proteinase K treatment can be left away
- shield - bud: 0 - 30 sec
- 1 somite: 30 - 60 sec
- 5 somites: 2 - 3 min
- 24 h embryos: 3 - 4 min
- 36 h-72 h embryos: 5 - 10 min at 2x the concentration (=10µg/ml)

3. **Rinse twice quickly with PBT** and then one PBT wash for 1 min followed by another PBT wash for 5 min to stop the digest.

4. Refixation in 4% PFA in PBS, 20 min at room temperature.

5. Wash in PBT, 5 times each 5 min (5 x 5 min).

6. Prehybridization:

Incubate embryos in 300 ml of Hyb buffer, 2 to 5 hrs at hybridization temperature (here: 67°C) in a water bath. The water bath should be warmed before. For shorter probes (300-500bp) one can go to 55-60°C in the water bath.

7. Hybridization:

Prepare the hybridization mixes (=probes in Hyb buffer)

The concentration of the probes depends on the kind and quality of probes and on the stage of the embryos.

Examples: vasa-Dig for 24h embryos: 0,5μl probe in 200μl of Hyb buffer.

vasa-Dig for sphere stage: 0,2μl probe in 200μl Hyb buffer.

myo D-Fluo, for second step in red, for 5s embryos: 2μl probe in 200μl Hyb buffer.

Heat the hybridization mix to 67°C (water bath) for ca. 10 min.

Remove prehybridization mix and replace with 200 μl of the preheated hybridization mix.

Hybridize overnight at 67°C (water bath).

<u>In situ Day 2:</u>

1.Washes:

Leave the tubes with embryos in the water bath. They can usually be left open (Close the lid of the water bath!). Always preheat the washing solutions to hybridization temperature.

Pipetting: with 10 ml pipette like before (Make sure that the pH of SSC 2x and 0.2x is 7.0).

- You can recover the probes: they can be used again (Sometimes they get better)
- **1**: Hyb wash (without tRNA) 500μl each tube, at 67°C, 20 min
- **2**: 50% SSCT 2 x / 50% Formamide, at 67°C, 3x 20 min
- **3**: 75% SSCT 2 x / 25% Formamide, at 67°C, 20 min
- **4**: SSCT 2x at 67°C, 2x 20 min
- **5**: SSCT <u>0.2 x</u> + 4 x 30 min, at 67°C
- **6**: PBT, 67°C, 5 min

2: blocking:

- 5% sheep serum
  + 10mg/ml BSA in PBT

incubate at room temp., several hrs (minimum 1 h). Put tubes on shaker or wheel (slow agitation) and keep dark (under aluminum), as Fluorescein-probes are being used.

3: Antibody-incubation I:

Incubation with anti-Dig-AP antiserum, preabsorbed against embryos, 1:2000- 1:4000 in PBT+2 mg/ml BSA (optional: +2% sheep serum). Keep the antibody at this concentration at 4°C. For storage, add Sodium Azide to 0,02%.

Incubate in 300 μl antibody solution overnight at 4°C without agitation (wrap in aluminum if Fluorescein-probes are being used). Incubation over 2 days at 4°C without agitation is also possible. During this incubation we lay the tubes on their sides.

In situ Day 3:

1. Washes after antibody:

Recover the antibody!! It can be reused several times.

Wash in PBT, at room temperature on shaker/ wheel etc (slow agitation), keep dark!

- 2 brief washes (5 min. each)
- 6-8 washes distributed over a total time of ca 3h.

2. **NTMT washes** (always prepare fresh!)

concentrations:	*amounts for 20ml:*
-100 mM Tris HCl pH 9.5,	2ml Tris HCl pH 9.5, 1M
-50 mM $MgCl_2$,	1ml $MgCl_2$, 1M
-100 mM NaCl,	2ml NaCl, 1M
-0.1% Tween 20	200 µl Tween 20, 10%
	14.8 ml H_20

Wash 3 x 5 min in NTMT. During last wash, transfer embryos to a 24 well plate.

For transfer of the embryos, use a 1ml pipette with cut tip. (The embryos stick to a glass pipette!)

3. Staining reaction:

Incubate embryos in staining solution at room temp. or in a 37°C incubator, in the dark, without shaking, and monitor the staining reaction from time to time using a dissecting microscope. Try to minimize the time you expose the embryos to light to avoid getting the background.

Staining solution BCIP/ NBT (blue staining):

3,5 µl NBT (Nitro Blue Tetrazolium, Sigma N6876, 75 mg/ml in 70% DMF / 30% H_2O)

3,5 µl X-phosphate (=BCIP) (50 mg/ml in 100% DMF)

1 ml NTMT buffer

For a 24 well plate, calculate 0.5 ml of staining solution per well.

The staining reaction can be interrupted by exchanging the staining solution for NTMT buffer. Like this, the samples can be kept at 4°C overnight. Next day: add newly made staining solution.

For slowly staining samples: Staining at 4°C overnight is also possible. Next day: go on staining at room temperature.

4. Stop the reaction by removing the staining solution and washing the embryos 3x in PBT.

To avoid yolk from becoming brown: wash and store in STOP solution. Just before you want to look at the embryos, put them into 80% glycerol + 20% STOP solution.

For one-color staining, after this step you can do the Clearing (go to Day 4, step2) and then store the embryos at 4°C.

In situ Day 4:

1. Removal of the first antibody:

- transfer embryos to 1.5 ml tubes
- Incubate embryos 2 x 5min in 0,1 M Glycin/HCl, pH 2.2 +0.1% Tween. Shake well!
- Wash 4 x 5 min in PBT.

2. (optional) Clearing:

If the yolk turned brownish during the staining procedure with NBT/ X-phosphate, clearing with EtOH gives very good results. After a few days of storage this brown precipitate would turn into a black, stable precipitate that cannot be dissolved away. Therefore, this EtOH treatment has to be done soon after the completion of the staining reaction. This step cannot be performed after the red staining reaction. (The red stain dissolves in ETOH very fast).

- incubate embryos in 100% EtOH, ca. 10 min or longer at room temp. (add EtOH, 100% directly, exchange 1x after ca. 5 min.). With slow or without agitation.
- 75% EtOH, 5 min.
- 50% EtOH, 5 min.
- 25% EtOH, 75% PBT, 5 min.
- PBT, 4 x 5 min.

3. Antibody-incubation II:

anti-Fluo-AP, preabsorbed against embryos, 1:2000- 1:4000 in PBT+2 mg/ml BSA (optional: +2% sheep serum). Keep the antibody at this concentration at 4°C. For storage, add Sodium Azide to 0,02%.

Incubate in 300µl overnight at 4°C without agitation, keep dark. During this incubation we lay the tubes on their sides.

No additional blocking step is required prior to the second antibody incubation.

In situ Day 5:

1. Washes after antibody:

Recover the antibody!! It can be reused several times.

Wash in PBT, at room temperature on shaker/ wheel etc (slow agitation), keep dark!

- 2 brief washes (5 min. each)
- 6-8 washes distributed over a total time of ca 3h.

- Wash 3 x 5 min in NTMT (freshly prepared). During last wash, transfer embryos to a 24 well plate. For transfer of the embryos, use a 1ml pipette with cut tip.

2. Staining reaction:

- Staining solution BCIP/ INT (red staining):
  3,5 µl INT (Sigma; 50 mg/ml in DMSO)

  3,5 µl X-phosphate (=BCIP) (50 mg/ml in 100% DMF)

  1 ml NTMT buffer

- Incubate in 0,5 ml staining solution per well, at room temp. or at 37°C. Keep dark.

3. Stop the color reaction by washing 3x in PBT and adding STOP solution.

4. Transfer embryos soon to 6-well plates in 80% Glycerol – 20% H_2O.

Solutions/ materials:

- X-Phosphate = BCIP
  Stock: 50mg/ml in 100%DMF. Store at –20°C.

- NBT
  Stock: 75 mg/ml in 0.7 ml of DMF+ 0.3 ml H_2O. Store at –20°C.

- INT (4-Iodonitrotetrazolium violet; from Sigma)
  Stock: 50 mg/ml in DMSO. Store at –20°C.

- PBS 10x 1l (*Gilbert*)
  -80 g NaCl

  -2 g KCl

  -18 g Na_2HPO_4 (2 H_2O)

  -2,4 g KH_2PO_4

  adjust pH to ca pH 7,2. Do not autoclave.

- PBT 1l
  -100 ml 10x PBS

-900 ml H_2O

-adjust the pH to 7,4 (usually by adding a few drops of 15% HCl)

+ 10 ml Tween 20, 10%

20x SSC

for 8 liter

1400gr NaCl

704gr TriNaCitrat 2•H20

adjust PH to 7.0 with HCl

for 1x SSCT add 0.1% tween 20

- Hyb buffer:
  for 1L:

  -250 ml SSC, 20x =5x (final concentration)

  -500 mg Torula yeast RNA* (Sigma) =500µg/ml

  -50 mg Heparin** = 50µg/ml

  -10 ml Tween 20, 10% *** = 0.1%

  -1,89 g Citric Acid (Monohydrate) = 9mM to pH 6.0-6.5

  - H_2O to 500ml

  -last: 500 ml Formamide, **deionized**. = 50%

  *tRNA is torula yeast RNA Sigma R6625 (50mg/ml).

  To make it Rnase free: 2x {0.5gr tRNA in 20ml 10x SSC} extracted with PCI and CI. follow the protocol for purifying sense or antisense RNA. After 2x wash with 80% EtOH, air dry and resuspend in 10x SSC

  Final concentration should be 500µg/ml in 10x SSC.

  ** heparin: (sigma H3393, 5mg/ml)

  *** Tween 20 (sigma P1379)

The final pH of Hyb buffer should be 6,0 – 6,5 (usually no pH adjustment is required). Store at –20°C.

- 0,1 M Glycin HCl, pH 2,2 + 0,1% Tween:
  for 0,5 l:

  -3,75 g Glycin

  -add H_2O to 400 ml

  -adjust pH to 2,2

  -fill to 500 ml with H_2O.

  -+ 5 ml Tween, 10%

  Store at room temp. After a few weeks or months, a white precipitate will form. Make a new solution.

- STOP solution:

  0.05M phosphate buffer pH 5.8

  1mM EDTA

  0.1% Tween

  for 200ml: 92ml 0.1M NaH2PO4

  8ml 0.1M Na2HPO4
  (this mixture should produce a pH of 5.8)

- **Preabsorption of the antibody**:

Method 1

-harvest hundreds of embryos, at different stages.

-fix overnight in 4% PFA in PBS.

-transfer to MetOH, 4-5 changes. Store embryos at –20°C.

-For preabsorbtion, take out about 200 embryos (for ca. 20 ml of final 1: 2000 antibody solution).

-wash embryos 2x in PBT and dechorionate by hand.

-wash another 5x in PBT.

-spin the concentrated antibody 5 min. at 13000 rpm.

-incubate antibody at a dilution of 1:400 on the embryos in BSA, 2mg/ml in PBT. Shake at room temp. for at least 1h. (The embryos can also be smashed prior to incubation. Spin down after incubation and carefully transfer the supernatant to a separate tube before diluting to the final concentration)

-Sterile filter using a 0,2 – 0,45 µm Cellulose Acetate filter. Best is dilute the antibody to 1: 2000 prior to filtering.

- final antibody solution:

1:2000 – 1:4000 in PBT, (optional: +2% sheep serum), +2mg/ml BSA.

- -Keep the antibody at this concentration at 4°C. For storage, add Sodium Azide to 0,02%.

Method 2

Process 50 embryos along with the others (same age) through all the first steps of in situ hybridization except that no probe is used for hybridization of these embryos.

during blocking of the others:

-block the embryos for preabsorption only 10 min.

-spin the concentrated antibody 5 min. at 13000 rpm.

-incubate antibody at a dilution of 1:400 on the embryos in BSA, 2mg/ml in PBT at RT for a few hours.

-remove antibody solution from the embryos, dilute to final concentration (as above; no sterile filtration required)

- SSC, 20x
- SSCT, 2x
- SSCT, 0,2x

Preadsoption of Abs

Prepare the anti X-AP antibody.

- Make 1:200 dilution of the Ab in blocking solution and pre adsorb it against fixed >MeOH-zebrafish embryos at different stages (primarily 24hrs) over night at 4 degrees on a wheel. (the embryos have to be treated the way those in a real in situ are treated i.e,

Prot.K, further fix and blocking.

- Fixed embryos should comprise approximately 10 % of the total volume.

- Use pre- adsorbed Ab at 500 to 5000 dilution (try 2000 to begin with)

Supplementary Method 2

Detailed RNAscope protocol for detection of transcripts in whole-mount zebrafish embryos, See Reference 31 for more details.

This protocol includes a step-by-step procedure for use in the laboratory.

**Overview**	The RNAscope Multiplex Fluorescent Assay for whole-mount zebrafish embryos can be completed within 2 days, allowing the detection of up to 4 different mRNAs simultaneously in different colors in combination with protein fluorescence.

Before the assay

* Unless otherwise mentioned, 0.01% of Tween concentration is used throughout the protocol (in PBT and 0.2X SSCT buffers).

**Fixation**	➢ Dechorionate embryos manually in PBT (0.1x Tween) in a Petri dish. After dechorionation transfer the embryos to 1.5ml Eppendorf tubes using a glass Pasteur pipette. For embryos younger than 24 hpf, first fix and then dechorionate. 24-hpf embryos and older were fixed without the chorion.<br><br>➢ Fix embryos at RT in 1ml freshly prepared or freshly thawed 4% PFA in PBS with the tube positioned on its side.<br><br>Fixation time:<br>4-cells to 8 hpf: 4 hours<br>12 to 20 hpf: 1 hour<br>24 hpf to 4 dpf: 30 minutes<br><br>The optimal fixation times for different stages and specific probes can be further optimized if needed.<br><br>➢ Remove the fixation solution and wash 3x5 minutes in 1ml PBT (0.1x Tween) at RT.<br><br>➢ Dehydrate embryos through a series of 25%, 50%, 75% MeOH in PBT (0.1x Tween) for 5 min each.<br><br>➢ Transfer embryos to 100% MeOH for 5 min, replace it with fresh MeOH and store at -20°C for overnight or longer.
**Prepare**	➢ Prepare 0.2X SSCT as the main wash buffer.

**wash buffers**	➢ Prepare 1X PBT.
**Heat Water bath**	➢ Heat the water bath to 40°C.
**Mix Target probes**	➢ Warm the probes at 40°C in the water bath for 10 min to dissolve precipitation and then bring it to RT.<br>➢ Spin down briefly the C2 and C3 probes to bring down the contents from the cap.<br>➢ Mix well the target probes of C1, C2 and C3 in a tube at 50:1:1 ratio. Use a final volume of 50-100µl per tube.
**Reagents to RT**	➢ Transfer all reagents from 4°C to RT.

The RNAscope detection step

**1. Drying**	➢ Remove the 100% MeOH completely from the tube of embryos.<br>➢ Let the embryos air-dry at RT for 30 min.
**2. Protease digestion**	➢ Add 2 drops of Pretreat 3 and incubate at RT for 20 min<br>➢ During the incubation position the tubes horizontally with very slow agitation to ensure homogenous treatment of the embryos.
**3. Stop digestion**	➢ Remove the Pretreat 3 solution.<br>➢ Rinse the embryos 3x 1ml PBT at RT.
**4. Probes hybridization**	**NOTE:**<br>• Pre-mixed probes should be pre-warmed to 40°C and then cooled down to RT before use.<br>➢ Add 50-100µl of mixed target probes per tube.<br>➢ Incubate overnight at 40°C.

	In case of high background and no need for fluorescent protein detection, use 50ºC.
**5. Probes removal**	➢ Recover the probes in a new tube. The recovered probes can be reused.<br><br>➢ Wash the embryos 3x 15 minutes with 1ml of 0.2X SSCT at RT.
**6. Postfixation**	➢ Fix the embryos again in 1ml of 4% PFA in PBS at RT for 10 min.<br><br>➢ Lay the tube on its side.
**7. Wash**	➢ Wash 3x 15 minutes with 1ml 0.2X SSCT at RT.
**8. Preamplifier hybridization**	➢ Remove the SSCT and replace it with 2 drops of Amp1. Gently tap the tube to mix completely.<br><br>➢ Incubate the embryos at 40°C for 30 min.
**9. Wash**	➢ Wash 3x 15 minutes with 1ml 0.2X SSCT at RT.
**10. Signal enhancemen t**	➢ Aspirate the SSCT and add 2 drops of Amp2. Gently tap the tube.<br><br>➢ Incubate the embryos at 40°C for 15 min.
**11. Wash**	➢ Wash the embryos 3x 15 minutes with 1ml 0.2X SSCT at RT.
**12. Amplifier hybridization**	➢ Aspirate the SSCT and replace it with 2 drops of Amp3. Tap the tube mildly.<br><br>➢ Incubate at 40°C for 30 min.
**13. Wash**	➢ Wash 3x 15 minutes in 1 ml 0.2X SSCT at RT.
**14. Label probe hybridization**	➢ Add 2 drops of Amp4 and tap the tube mildly and incubate at 40°C for 15 min. Using Amp4 alternative solutions (AltA, B or C) different probe and fluorophore combination is possible. (See also Table 4)
**15. Wash**	➢ Rinse 3x 15 minutes with 1ml 0.2X SSCT at RT.

**16. Counter stain**	➢ Remove the SSCT and add 2 drops of DAPI per tube or Hoechst: 0.2X SSCT at a 1:10000 ratio.<br><br>➢ Incubate overnight at 4°C with slow agitation.
**17. Preparation for microscopy**	➢ Rinse the embryo with 1ml 1X PBT and then prepare for imaging using 1% LMP in a Petri dish.<br><br>➢ Fill the Petri dish with 1X PBS to allow imaging using water-immersion objectives.
**18. Microscopy**	➢ Image the samples using a fluorescent confocal microscope.<br>See Table 4 for filter set specification for microscopy.<br><br>➢ The embryos do not have to be "deyolked", since the presented RNAscope procedure produces minimal background fluorescence in the yolk.<br><br>➢ Wrap tubes in aluminium foil and store at 4°C for later imaging.

Supplementary References:

1. Dereeper, a. *et al.* Phylogeny.fr: robust phylogenetic analysis for the non-specialist. *Nucleic Acids Res.* **36,** 465–469 (2008).
2. Blaser, H. *et al.* Transition from non-motile behaviour to directed migration during early PGC development in zebrafish. *J. Cell Sci.* **118,** 4027–38 (2005).
3. Tarbashevich, K., Reichman-Fried, M., Grimaldi, C. & Raz, E. Chemokine-Dependent pH Elevation at the Cell Front Sustains Polarity in Directionally Migrating Zebrafish Germ Cells. *Curr. Biol.* **25,** 1096–1103 (2015).
4. Krens, S. F. G., Möllmert, S. & Heisenberg, C.-P. Enveloping cell-layer differentiation at the surface of zebrafish germ-layer tissue explants. *Proc. Natl. Acad. Sci. U. S. A.* **108,** E9–10; author reply E11 (2011).
5. Field, H. A., Si Dong, P. D., Beis, D. & Stainier, D. Y. R. Formation of the digestive system in zebrafish. II. Pancreas morphogenesis. *Dev. Biol.* **261,** 197–208 (2003).
6. Haas, P. & Gilmour, D. Chemokine Signaling Mediates Self-Organizing Tissue Migration in the Zebrafish Lateral Line. *Dev. Cell* **10,** 673–680 (2006).
7. Valentin, G., Haas, P. & Gilmour, D. The Chemokine SDF1a Coordinates Tissue Migration through the Spatially Restricted Activation of Cxcr7 and Cxcr4b. *Curr. Biol.* **17,** 1026–1031 (2007).
8. Stainier, D. Y. *et al.* Mutations affecting the formation and function of the cardiovascular system in the zebrafish embryo. *Development* **123,** 285–292 (1996).
9. van Eeden, F. J. *et al.* Mutations affecting somite formation and patterning in the zebrafish, Danio rerio. *Development* **123,** 153–164 (1996).
10. Chen, J. N. *et al.* Mutations affecting the cardiovascular system and other internal organs in zebrafish. *Development* **123,** 293–302 (1996).
11. Knaut, H., Werz, C., Geisler, R. & Nüsslein-Volhard, C. A zebrafish homologue of the chemokine receptor Cxcr4 is a germ-cell guidance receptor. *Nature* **421,** 279–82 (2003).
12. Jao, L. E., Wente, S. R. & Chen, W. Efficient multiplex biallelic zebrafish genome editing using a CRISPR nuclease system. *Proc. Natl. Acad. Sci.* **110,** 13904–13909 (2013).
13. Elgeti, J. & Gompper, G. Wall accumulation of self-propelled spheres. *Europhysics Letters* **101,** (2013).
14. Redner, S. A guide to first-passage processes. *Cambridge University Press* (2001).
15. Schnitzer, M. J. Theory of continuum random walks and application to chemotaxis. *Physical Review E* **48,** 2553-2568 (1993).
16. Tailleur J. & Cates, M. E. Statistical mechanics of interacting run-and-tumble basteria. *Physical Review Letters* **100,** (2008).
